# A review of applications of environmental DNA for reptile conservation and management

**DOI:** 10.1002/ece3.8995

**Published:** 2022-06-05

**Authors:** Bethany Nordstrom, Nicola Mitchell, Margaret Byrne, Simon Jarman

**Affiliations:** ^1^ School of Biological Sciences The University of Western Australia Crawley Western Australia Australia; ^2^ Department of Biodiversity, Conservation and Attractions Biodiversity and Conservation Science Perth Western Australia Australia; ^3^ UWA Oceans Institute The University of Western Australia Crawley Western Australia Australia

**Keywords:** biomonitoring, DNA metabarcoding, eDNA, invasive species, reptile diversity, threatened species

## Abstract

Reptile populations are in decline globally, with total reptile abundance halving in the past half century, and approximately a fifth of species currently threatened with extinction. Research on reptile distributions, population trends, and trophic interactions can greatly improve the accuracy of conservation listings and planning for species recovery, but data deficiency is an impediment for many species. Environmental DNA (eDNA) can detect species and measure community diversity at diverse spatio‐temporal scales, and is especially useful for detection of elusive, cryptic, or rare species, making it potentially very valuable in herpetology. We aim to summarize the utility of eDNA as a tool for informing reptile conservation and management and discuss the benefits and limitations of this approach. A literature review was conducted to collect all studies that used eDNA and focus on reptile ecology, conservation, or management. Results of the literature search are summarized into key discussion points, and the review also draws on eDNA studies from other taxa to highlight methodological challenges and to identify future research directions. eDNA has had limited application to reptiles, relative to other vertebrate groups, and little use in regions with high species richness. eDNA techniques have been more successfully applied to aquatic reptiles than to terrestrial reptiles, and most (64%) of studies focused on aquatic habitats. Two of the four reptilian orders dominate the existing eDNA studies (56% Testudines, 49% Squamata, 5% Crocodilia, 0% Rhynchocephalia). Our review provides direction for the application of eDNA as an emerging tool in reptile ecology and conservation, especially when it can be paired with traditional monitoring approaches. Technologies associated with eDNA are rapidly advancing, and as techniques become more sensitive and accessible, we expect eDNA will be increasingly valuable for addressing key knowledge gaps for reptiles.

## Graphical abstract

Although eDNA has had limited application to reptiles relative to other vertebrate groups, this review demonstrates its ability to inform on reptile distribution and trophic interactions. eDNA techniques have been more successfully applied to aquatic reptiles than to terrestrial reptiles. As technologies associated with eDNA advance and become more sensitive, it will become increasingly valuable for addressing key knowledge gaps for reptiles.

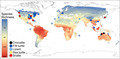

## 1 INTRODUCTION

Reptiles are a diverse group of tetrapods, with representatives in terrestrial and aquatic (freshwater and marine) habitats in temperate, tropical, and arid environments (Böhm et al., 2013). For the purpose of this review, we define reptiles according to the Linnaean classification system based on unique shared characteristics (such as covered scales, ectothermic physiology). This commonly accepted definition of reptiles is necessary as they are a paraphyletic group, not a true clade. Reptiles include animals from the following extant orders: Testudines, Rhynchocephalia, Squamata, and Crocodilia, encompassing all of the amniotes except birds and mammals.

Approximately 21% of reptile species are threatened with extinction (Cox et al., 2022), and the world's total reptile populations are estimated to have declined by 55% in the last 50 years (Saha et al., 2018). Primary drivers of decline include habitat loss, climate change, invasive species, and over‐harvesting (Böhm et al., 2013). Many reptile species have small native distributions and narrow thermal niches due to their ectothermic physiology, making smaller populations especially vulnerable to a range of common environmental pressures (Böhm et al., 2013). Conversely, several reptile species have been introduced beyond their indigenous ranges, and in many cases have impacted trophic dynamics and decreased native species abundance (Kraus, 2015). Notably, two reptiles are among the “top 100” of the world's most disruptive invasive species; the brown tree snake (*Boiga irregularis*) and the red‐eared slider (*Trachemys scripta elegans*) (Lowe et al., 2000).

The geographical distribution of a species, trends in population size, and trophic interactions are essential parameters for informed management of threatened and invasive species (Saunders et al., 2018). Traditionally, this information is collected via physical sampling or observational surveys, which can be time‐consuming, invasive (e.g., requiring handling for collection of blood, tissue, or stomach content samples), and expensive (Beng & Corlett, 2020). Species traits, such as restricted daily activity, dispersal phases, and cryptic morphology, make many species of reptile difficult to detect (Lacoursière‐Roussel et al., 2016). Consequently, robust and noninvasive monitoring tools are needed to better document species distributions and population trends.

Non‐invasive molecular approaches to ecological studies have become increasingly common over the last decade—in particular the use of environmental DNA (Taberlet et al., 2012). Environmental DNA (eDNA) is DNA that is extracted and identified using molecular approaches from an environmental samples (such as soil, water, sediment, air, or feces), (Taberlet et al., 2018). eDNA is purified from a complex mixture of different molecule types, both biological and geological, larger particles, living microorganisms and degraded components of macroorganisms. A variety of sampling procedures for eDNA exist that result in collecting different subsets of the environmental substrate. All eDNA analysis on the sampling material involves chemical and enzymatic manipulation to concentrate the true eDNA components from a background of complex molecules. The range of environmental substrates that the term “eDNA” refers to has expanded over time and now also includes any biological samples of mixed biological origin, such as fecal material (Pawlowski et al., 2020). eDNA analysis methods have piqued the interest of conservation biologists, ecologists, and managers—especially those interested in elusive, cryptic, or rare species. DNA from environmental samples is identified in two main ways: specific‐species detection using primers/assays specific to one species (often with quantitative PCR (qPCR)), and DNA metabarcoding to detect entire communities (Lopes et al., 2021; Mauvisseau et al., 2019; Tilker et al., 2020; Valdivia‐Carrillo et al., 2021) and conduct dietary analyses (Ando et al., 2020). DNA metabarcoding uses universal primers and next generation sequencing to maximize DNA detection for a wide range of target species (e.g., the 12S primer VERT01 targets all vertebrates (Taberlet et al., 2018)).

Detecting reptile DNA from environmental samples is reputedly difficult to achieve (Adams et al., 2019; Baker et al., 2020; Kucherenko et al., 2018; van der Heyde et al., 2021), especially when compared to other animal taxa such as fish or amphibians (Adams, Hoekstra, et al., 2019; Raemy & Ursenbacher, 2018). Here, we review literature on eDNA studies of reptiles and summarize the utility of eDNA in reptile conservation and management. We then focus on the benefits and limitations of using eDNA as an ecological indicator and identify research gaps and future directions of this molecular approach.

## 2 LITERATURE SEARCH

**FIGURE 1:**
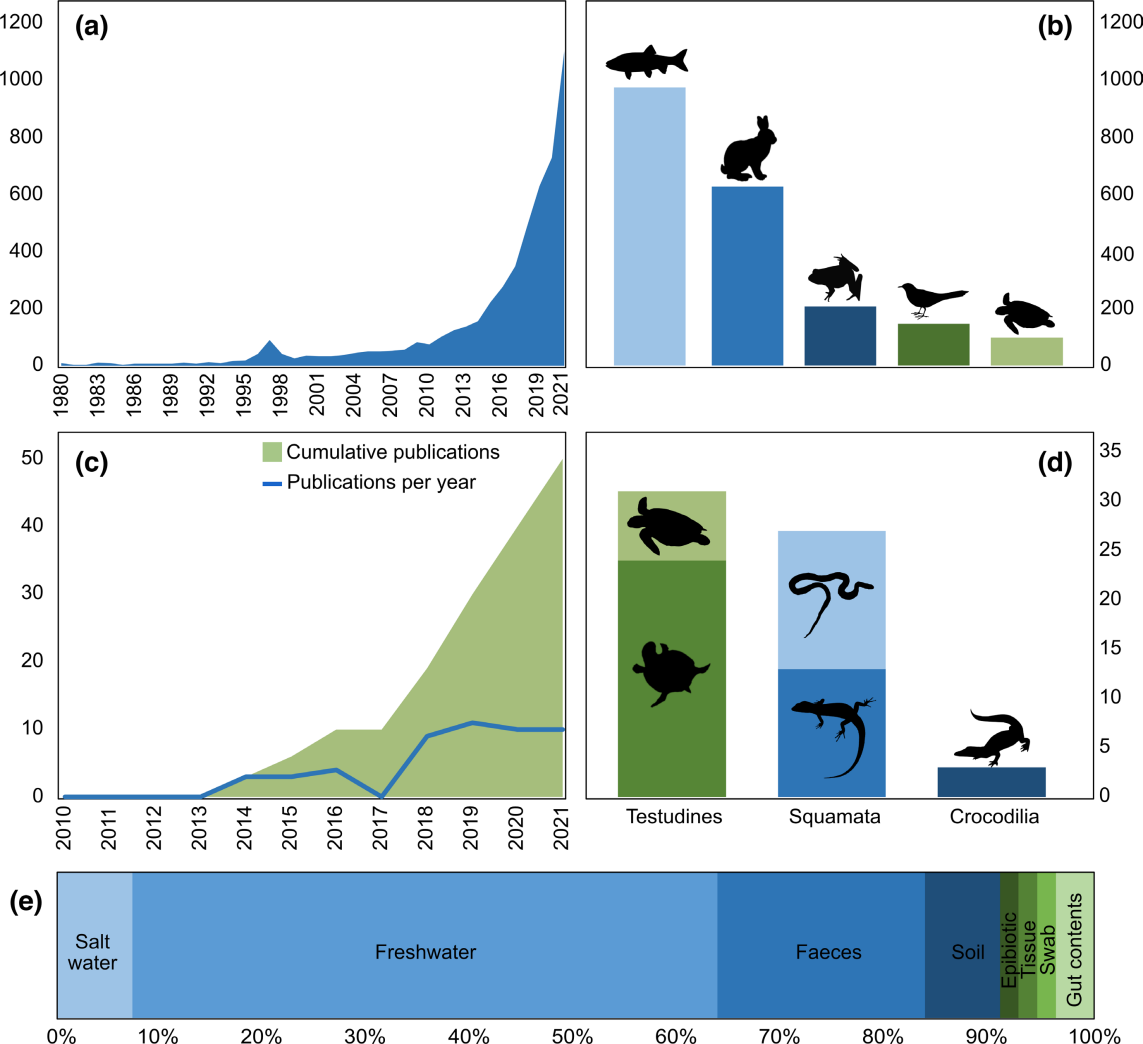
Literature search results: (a) eDNA publications per year from Scopus search: (“eDNA” OR “environmental DNA” OR “DNA metabarcoding”) in the title/abstract/keywords from 1980 to 2020; (b) Number of eDNA publication for the five main vertebrate groups: fish, mammal, bird, amphibian, and reptile; (c) Reptile eDNA publications per year (blue line) and cumulative publications from 2010 to 2020 (green); (d) Number of reptile eDNA publications broken into order, Testudines (light green = sea turtles, dark green = freshwater turtles), Squamata (light blue = snakes, medium blue = lizards), and Crocodilia (dark blue) (no publications existed for Rhynchocephalia); (e) Percentage of studies by sample type collected for eDNA analysis

To compare the use of eDNA across the five main vertebrate taxa (fish, mammals, birds, amphibians, and reptiles), literature searches were conducted in Scopus in March 2022 (see Panel S1 in Appendix S1). The literature searches resulted in 2061 publications, and reptiles were represented the least out the five vertebrate groups in the eDNA literature (Figure 1b). Fish were represented ten times more than reptiles, mammals six times, amphibians two times, and birds 1.5 times (Figure 1b). To evaluate the utility of eDNA in reptile conservation and management, the identified publications focused on eDNA and reptiles were examined further. The Scopus search yielded 99 results, and a secondary search was conducted in Google Scholar for any reptile eDNA studies that may have been missed in Scopus. These were then reduced to 55 peer reviewed articles after manually selecting those that met the criteria of an eDNA study with a primary focus on reptile ecology, conservation, or management (Figure 1c). The first reptile eDNA studies were published in 2014 (Brown et al., 2014; Kelly et al., 2014; Piaggio et al., 2014), with an increasing rate of publication since 2017 (Figure 1c). Adams, Hoekstra, et al. (2019) briefly reviewed reptile eDNA literature, which included 14 papers, and so the 55 published studies that now exist represent an approximately 400% increase in the literature and support the need for an updated review.

Of the 55 published studies, 31 focused on turtles (24 freshwater, seven marine), 27 on squamates (14 on snakes, and 13 on lizards), and three on crocodiles (Figure 1d) (note: as some studies focused on more than one group of reptile, the total of published studies and total studies by reptile group do not correspond). The focus of most studies (38 of 55) was determining the spatial distribution, ecology, and population trends of threatened species, followed dietary niche via DNA metabarcoding (16 of 55). Eleven papers focused on impacts of invasive species, and the remaining had other overarching themes including disease in reptiles, wildlife–human interactions, and defining ecological roles. Most studies sampled aquatic ecosystems (35 of 55), with fecal, soil/sediment, and epibiotic samples constituting the remainder (Figure 1e). Species‐specific detection was the most common approach (36 of 55), while DNA metabarcoding was used in 20 studies (16 of these being dietary studies) (see Table S1 in Appendix S1).

## 3 DISTRIBUTION of eDNA STUDIES

**FIGURE 2:**
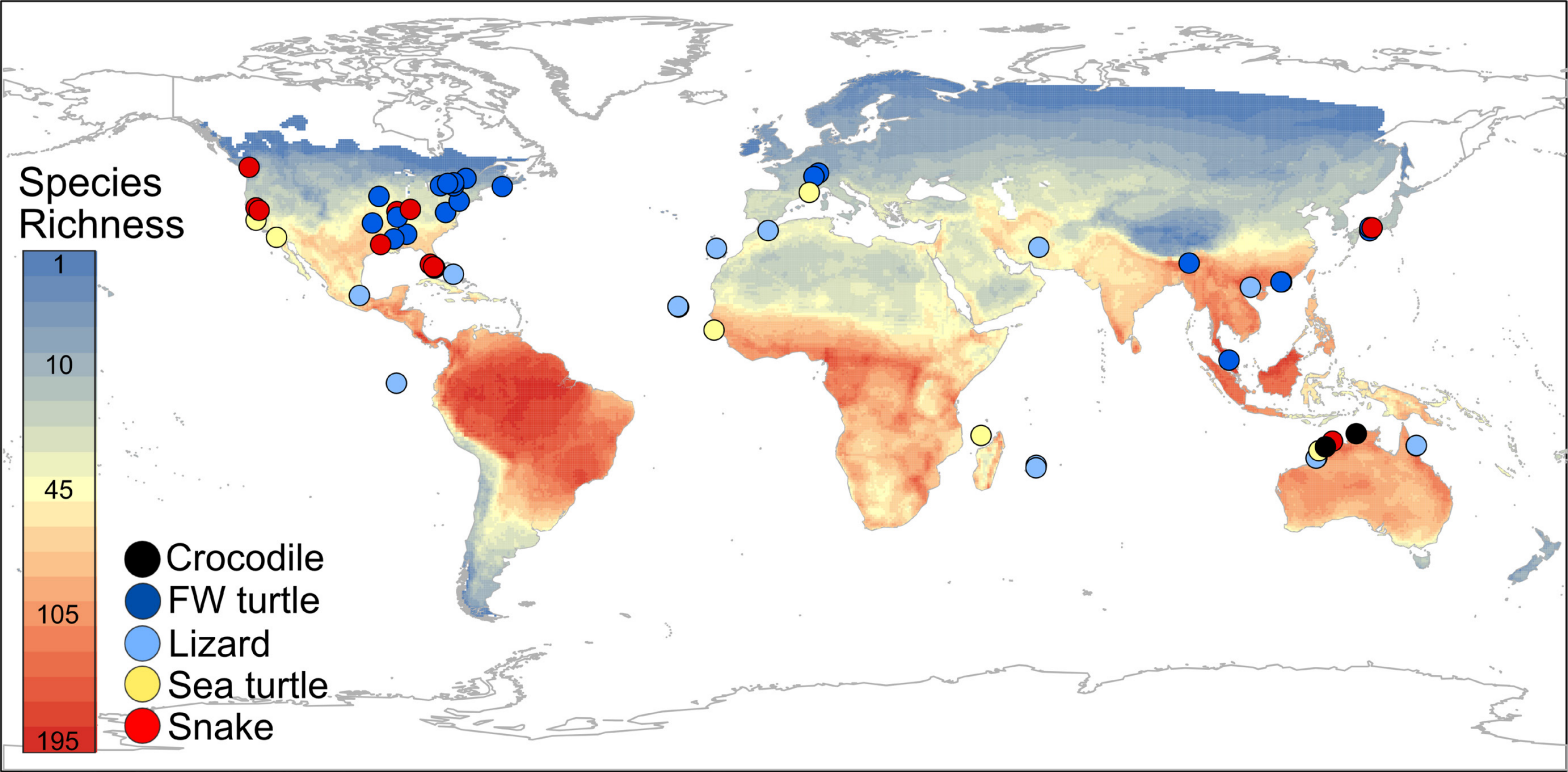
Locations of reptile eDNA samples collection, broken up into main groups: crocodilians (black circles), sea turtles (yellow circles), lizards (light blue circles), snakes (red circles), and freshwater turtles (dark blue circles). Study locations are superimposed onto a global map of reptile richness at 1 degree spatial resolution (Roll et al., 2017)

eDNA studies have mostly been conducted in North America (51% of studies, including 11 snake, 13 freshwater turtle, two sea turtle, and two lizard studies) (Figure 2). Studies incorporating eDNA are uncommon in areas of high reptile species richness, including South America, Sub‐Saharan Africa, Southeast Asia, and Australia (Figure 2) (Roll et al., 2017). eDNA metabarcoding is an approach that can detect many species in one assay (Table 1) and so is particularly useful in regions of high species richness. West et al. (2021) illustrates the utility of DNA metabarcoding to describe assemblages of aquatic reptiles across northern Australia, where nine aquatic reptile species were detected using a mitochondrial 16S assay designed for reptiles. Polanco et al. (2021) used a mitochondrial 12S assay (VERT01 (Taberlet et al., 2018)) to describe biodiversity in tropical Columbia in estuaries and marine waters. This primer was designed for all vertebrates, and successfully detected one species of reptile (the spectacled caiman, *Caiman crocodilus*) and two families of reptile (Alligatoridae and Kinosternidea), along with several amphibians, birds, mammals, and fish (Polanco et al., 2021).

**TABLE 1:** Definitions of technical terms used in the field of environment DNA

Term	Definition
Digital droplet PCR (ddPCR)	Allows for absolute quantification of target DNA, without a standard curve of the reference (Doi et al., 2015). This occurs by separating the PCR mixture into approximately 20,000 droplets via an oil emulsion, where a PCR reaction and amplification (using fluorescence) occurs in each droplet (Capo et al., 2019; Doi et al., 2015). The concentration of target DNA from the sample can be determined by end‐point analysis of the nanodroplets (Doi et al., 2015). Also referred to as “third‐generation PCR.”
DNA metabarcode	A region of DNA that varies between species in its central region, while having consistent ends that allow PCR amplification. The variable central region allows taxa to be differentiated and identified by reference to sequences of known taxonomic provenance. For single sequences corresponding to one species, this is called “DNA barcoding.”
DNA metabarcoding	Simultaneous taxonomic identification of multiple species or multiple groups (family, genus, etc.) within the same environmental sample. PCR primers designed to amplify DNA metabarcodes for certain groups are applied to eDNA samples using conventional PCR. The amplified DNA is sequenced by HTS. DNA metabarcoding is often used in biodiversity monitoring, and diet analysis.
High‐throughput sequencing (HTS)	HTS technologies (e.g., Illumia, IonTorrent, PacBio, Roche) produce multiple sequences in parallel, allowing millions of DNA molecules to be sequenced simultaneously. Also referred to as “next‐generation sequencing (NGS).”
Polymerase chain reaction (PCR)	A laboratory process used to make multiple copies (amplify) of small segments of DNA.
Primer	A short sequence of single‐stranded DNA (15 to 35 bases) that enables replication of DNA during the PCR process. Primers are designed to match a specific DNA template, and if they do not match, DNA polymerase will not bind and amplification will not occur.
Probe	A fragment of DNA used to detect a specific sequence in a sample, by binding with complementary bases of the target sequence. Labels are chemically attached to probes (radioactive or fluorescent material), allowing visualisation of binding.
Quantitative PCR (qPCR)	qPCR uses fluorescent dyes that bind to DNA as it amplifies. The fluorescent signal is measured after each PCR cycle, and a standard curve is constructed from the threshold per cycle (CT), allowing for quantification of the amount of DNA in the sample as the reaction proceeds.

Most data‐deficient reptiles (19% of all reptile species) occur in tropical areas such as Central Africa and Southeast Asia (Böhm et al., 2013), which are also hotspots of reptile species richness (Figure 2) (Roll et al., 2017). Areas of high reptile richness support many species threatened by climate change (Böhm et al., 2016), and hence it is especially critical to monitor their occurrence, distribution, and population dynamics in these regions. eDNA approaches (both species‐specific monitoring, and DNA metabarcoding for biodiversity sampling/dietary analysis) in areas of high reptile richness could help increase knowledge on data‐deficient and threatened reptile species and communities.

## 4 THREATENED SPECIES

Our literature search indicates an emerging importance of eDNA in reptile conservation and management, with ~70% of papers focused on threatened species, ~18% on invasive species, and ~65% using species‐specific detection to investigate species distribution and occurrence (Table S1 in Appendix S1). More than half of the studies involved Testudines (turtles and tortoises)—one of the most threatened groups of vertebrates with >50% of extant species at risk of extinction (Rhodin et al., 2018) and peak species richness in south eastern USA and South East Asia (Roll et al., 2017). Sample collections for nine of the 24 freshwater turtle studies occurred in these regions, while another eight came from north eastern USA and southern Canada (Figure 2).

Many studies focused on the detection of threatened species, particularly in demonstrating the effectiveness of eDNA to complement traditional survey methods. Conservation legislation in certain countries requires critical habitat for threatened species to be protected (e.g., Canada [Species At Risk Act, 2002]); and for such protection to occur, the presence of the threatened species should be confirmed. eDNA sampling enables cost‐effective and efficient time‐sensitive monitoring at varying spatial and temporal scales (Beng & Corlett, 2020; Ficetola et al., 2019; Reinhardt et al., 2019), and as sampling is noninvasive and nondestructive, it is ideal for sampling sensitive habitats or highly threatened species (Beng & Corlett, 2020). Davy et al. (2015) developed species‐specific primers for nine freshwater turtle species in Canada, where traditional sampling for the turtles is time‐consuming and not always successful. After successfully detecting all nine species with eDNA in controlled settings, Davy et al. (2015) proposed that eDNA could be used as a preliminary survey method to sample potential turtle habitat. Areas with positive eDNA detections could then be rigorously sampled using traditional methods paired with eDNA methods.

## 5 INVASIVE SPECIES

Detecting rare, cryptic, and secretive reptiles with eDNA methods is also relevant for invasive species (Larson et al., 2020). For example, eDNA can detect invasive species at the boundaries of their current distribution, where their density may be low and range expansion may be occurring (Hunter et al., 2019; Larson et al., 2020; Valentin et al., 2018). A relevant case study is the Burmese python (*Python bivittatus*), which are native to Southeast Asia, but first detected in southern Florida in the 1990s (Dorcas et al., 2012; Piaggio et al., 2014). Pythons are slow‐moving, semi‐aquatic, and cryptic, and have a very low detection rate of 0.05% per trap night in the Florida Everglades (Hunter et al., 2015). A PCR primer test successfully detected pythons in both a controlled environment, and in the field (Piaggio et al., 2014). Building upon this, Hunter et al. (2015) developed a species‐specific primer using qPCR, recognized for its high specificity, sensitivity, and reduction in false positives (Nathan et al., 2014; Wilcox et al., 2013). Burmese pythons were then detected at the northern edges of their known distribution (Hunter et al., 2015). The qPCR assay was adapted for use with ddPCR (digital droplet PCR—which is regarded as being more sensitive than qPCR (Mauvisseau et al., 2019)), revealing a high occurrence of positive eDNA detections further north than the established population boundary (Hunter et al., 2019). This case study demonstrates the utility of eDNA for monitoring range limits and expansion of invasive species, and as a method for assessing the effectiveness of control efforts.

## 6 COST‐EFFECTIVENESS OF eDNA METHODS

eDNA sampling is recognized as being a cost‐effective alternative to traditional sampling and intensive field surveys (Fediajevaite et al., 2021). Davy et al. (2015) estimated traditional survey methods to detect freshwater turtles cost between two and ten times more than eDNA. Another study compared the cost of detecting threatened wood turtles (*Glyptemys insculpta*), finding that traditional visual encounter surveys were between two and six times more expensive than eDNA surveys (Akre et al., 2019). One reason for this cost‐effectiveness is that eDNA sampling generally requires fewer person‐hours than methods of traditional sampling, such as visual encounter surveys and trapping surveys (Davy et al., 2015; Mena et al., 2021). Time and cost benefits when using eDNA surveying over traditional surveying can be considerable when working with rare, cryptic, or threatened species. However, several factors influence costs associated with eDNA sampling, including those associated with setting up an eDNA facility, validating and troubleshooting new assays, the number of primers used, reagents, and sequencing depth for metabarcoding approaches so applications need to be considered in context (Ficetola et al., 2019). Only two reptile studies provided a quantitative comparison of costs between the two methods (Akre et al., 2019; Davy et al., 2015), and due to high variability in costs associated with individual projects, it is not yet possible to conclude that eDNA methods are cheaper.

## 7 INTEGRATING eDNA AND OTHER SURVEY METHODS

While eDNA sampling may offer time and cost benefits over traditional survey methods, it is often acknowledged that pairing eDNA with another survey method likely results in the best outcome (Adams, Hoekstra, et al., 2019; Raemy & Ursenbacher, 2018; Rose et al., 2019). A recent meta‐analysis revealed that while eDNA outperforms traditional surveys for most taxa, it was less sensitive than traditional surveys for detecting reptiles (Fediajevaite et al., 2021). Fediajevaite et al. (2021) recognized that this may partially reflect research effort, as reptile studies were the second least represented in the meta‐analysis. Of the studies reviewed here, only 22% compared eDNA methods and traditional surveying (with temporal overlap between the two methods), with mixed results: three found eDNA and traditional survey detections were comparable (Akre et al., 2019; Kakuda et al., 2019; Kucherenko et al., 2018), three found eDNA outperformed traditional surveys (Feist et al., 2018; Matthias et al., 2021; Raemy & Ursenbacher, 2018), and two found that traditional surveys outperformed eDNA (Ratsch et al., 2020; Rose et al., 2019). These studies were not able to be directly compared to conclude whether eDNA outperforms traditional surveying due to substantial variation in sample methods, design, and reporting metrics.

eDNA methods can also be strengthened by pairing with site‐occupancy modeling (Burian et al., 2021; Schmidt et al., 2013)—an approach taken in several reptile studies (Akre et al., 2019; de Souza et al., 2016; Hunter et al., 2015, 2019; Kessler et al., 2020; Lacoursière‐Roussel et al., 2016; Orzechowski et al., 2019; Rose et al., 2019). Occupancy models determine the probability of true species presence or absence at a site, and can account for imperfect detections in ecological surveys (MacKenzie et al., 2002). In brief, repeated surveys are taken at each site, resulting in a series of detection/nondetections. An occupancy model calculates a certainty estimate that a site is unoccupied, given no detections (Rose et al., 2019). Imperfect detections in eDNA studies come from either field sampling or the laboratory analysis. A negative eDNA detection does not necessarily mean the species is absent, but occupancy models can help estimate the false‐negative rate, which is particularly important when working with threatened or invasive species.

While occupancy modeling has only been applied to a small fraction of the species‐specific detection studies in this review (7/36), in each case the results have provided valuable context to help better understand eDNA occurrence and detection. Some of these extend beyond providing simple occupation and detection probabilities at a given site for the species of interest. For example, occupancy modeling has been used to estimate appropriate sample size (Akre et al., 2019; Hunter et al., 2015), the minimum number of replicated (de Souza et al., 2016) and to determine other biotic (i.e., biomass) and abiotic factors (i.e., UV exposure, temperature, seasonality) that affect eDNA (Akre et al., 2019; de Souza et al., 2016; Kessler et al., 2020). Finally, occupancy modeling has been used to directly compare traditional survey methods with eDNA sampling. Rose et al. (2019) found that traditional sampling methods had higher detection probabilities than eDNA methods, while Akre et al. (2019) found comparable occupancy and detection probabilities between traditional visual encounter surveys and eDNA. Given this uncertainty surrounding the effectiveness and reliability of eDNA as a survey method for reptiles, occupancy models should also be used when possible, as they provide quantitative values for imperfect detections and interpreting eDNA results, information on appropriate sampling regimes, and a better understanding of environmental and habitat covariates.

## 8 USE OF eDNA IN DIETARY ANALYSIS

Defining trophic interactions and trophic niches are valuable for both ecosystem and single‐species management (de Souza et al., 2016). Traditionally, animal diets are investigated by visually examining stomach contents and/or fecal samples, or using stable isotope analysis (Nielsen et al., 2017). These methods often require one or more taxonomic experts, can be invasive (e.g., stomach flushing), or prey may be highly digested. Such limitations result in uncertainty in identifying trophic niches, and species interactions in food webs. For example, the trophic niches of many lizard species have not been defined, partly due to challenges in identifying prey taxa in fecal pellets (Pereira et al., 2019). DNA metabarcoding of fecal samples can identify prey at a high taxonomic resolution and reveal previously unknown aspects of species diet (Gil et al., 2020; Jarman et al., 2013; Lopes et al., 2019). Using DNA metabarcoding, Pinho et al. (2018) showed that the Endangered giant wall gecko (*Tarentola gigas*) predates one of the world's rarest bird species, the Raso lark (*Alauda razae*) in the Capo Verde Archipelago off the west coast of Africa. Not only was predation occurring, the Raso lark was the most frequent vertebrate signature found in the gecko's feces (Lopes et al., 2019), creating an interesting conservation dilemma in relation to management of these two threatened species. In another study, Gil et al. (2020) used DNA metabarcoding to show that a gecko previously assumed to be insectivorous, was actually a generalist. This resulted in a greater understanding of its role in the ecological network (Gil et al., 2020). Hence DNA metabarcoding, especially when used for dietary analysis, shows great promise for effective identification of trophic niches and reconstruction of food webs (Ficetola et al., 2019)—aspects of reptile ecology that are often poorly understood.

## 9 AQUATIC VS. TERRESTRIAL REPTILES

While only ~8% of living reptiles are partially or wholly aquatic (Thewissen & Nummela, 2008), more than 60% of the reptile eDNA studies sampled aquatic environments (Figure 1e). This is not surprising as water as a sampling medium for eDNA studies is well established and is highly successful (Beng & Corlett, 2020; Ruppert et al., 2019).

Unsurprisingly, species‐specific eDNA approaches for terrestrial‐based reptiles were highly underrepresented in the literature: only four studies analyzed soil samples (Figure 1e; Table S1 in Appendix S1). Kucherenko et al. (2018), Katz et al. (2021), and Matthias et al. (2021) were able to detect the presence of snake eDNA in soil samples in the field. In comparison, Ratsch et al. (2020) was unable to detect Kirtland's snake in any soil samples. DNA fragments can persist longer in soil (up to decades to centuries in some circumstances) relative to aquatic settings (freshwater = days to weeks), which makes it difficult to determine whether eDNA from soil samples reflect the current ecosystem (Foucher et al., 2020; Taberlet et al., 2018). Soil samples also tend to have high levels of humic substances, which can negatively impact PCR amplification through inhibition and result in false negatives (Thomsen & Willerslev, 2015). However, there are many examples of successful eDNA studies that use soil samples; notably for fungi (Buée et al., 2009; Rosa et al., 2020) and plants (Drummond et al., 2015; Foucher et al., 2020; Taberlet et al., 2012), but also for earthworms (Bienert et al., 2012), birds (Drummond et al., 2015), and mammals (Andersen et al., 2012; Leempoel et al., 2020). These diverse soil‐based studies might suggest that the few reptile examples to date do not reflect the capacity for use of this technique for terrestrial reptile studies.

eDNA methods for detecting terrestrial reptiles are not limited to soil and sediment samples. Several recent studies have shown the potential of using water samples to detect terrestrial mammals (Harper, Griffiths, et al., 2019; Lyet et al., 2021; Mas‐Carrió et al., 2021; Mena et al., 2021), and birds (Mas‐Carrió et al., 2021). Terrestrial animals visit water bodies, and their DNA can be transferred to aquatic systems directly through behaviors such as foraging, drinking, swimming, defecation, and bathing, or indirectly via rain and soil drainage (Coutant et al., 2021). Mas‐Carrió et al. (2021) used the 12S primer VERT01 (Taberlet et al., 2018) to target terrestrial birds, reptiles, and mammals in remote desert water bodies, although they did not detect any reptiles through eDNA or through camera trap surveys.

Air sampling has recently shown potential to detect terrestrial animals (Clare et al., 2022; Lynggaard et al., 2022; Roger et al., 2022). Lynggaard et al. (2022) collected eDNA from air using water‐based commercials vacuums and air particle filters in several locations at Copenhagen Zoo, Denmark, including a Tropical house that contained reptiles, birds, and mammals. One species of reptile, Dumeril's ground boa (*Acrantophis dumerili*), was successfully detected using the 12S primer 12SVO5 (Riaz et al., 2011), along with 16 mammal, eight bird, three fish, and one amphibian species (Lynggaard et al., 2022). Sampling surface substrates has also shown potential for surveying terrestrial animals (Valentin et al., 2020). One method described as “tree rolling” uses sterile cotton rollers to collect eDNA from trees (Valentin et al., 2020), and could be explored for detection of arboreal reptiles.

## 10 QUANTIFYING ABUNDANCE/BIOMASS WITH eDNA

Monitoring population dynamics is key for effective species management (Ficetola et al., 2019), but the utility of eDNA as a quantitative tool for estimating biomass or individual numbers is a source of debate (Capo et al., 2019). While there are semi‐quantitative approaches, such as frequency of occurrence or relative number of sequences (usually associated with DNA metabarcoding) (Deagle et al., 2019), no current eDNA technique can estimate absolute abundance (Yates et al., 2019). Some studies using qPCR show positive correlations between abundance and eDNA concentrations or amplifications, including those on reptiles (Adams, Knapp, et al., 2019; Kakuda et al., 2019; Lacoursière‐Roussel et al., 2016), but others show no correlation (Raemy & Ursenbacher, 2018). Estimates of abundance using eDNA need to be interpreted with caution, as eDNA concentrations can be influenced by a variety of individual traits (e.g., metabolic activity, body size), environmental factors (e.g., temperature, UV levels, physical transport), and technical considerations (e.g., number of PCR cycles, primer design, inhibitors) (Beng & Corlett, 2020; Capo et al., 2019; Ficetola et al., 2019). Importantly, metabolic rate and activity in ectotherms such as reptiles are driven by environmental temperatures, which may influence eDNA concentrations (Beng & Corlett, 2020; Lacoursière‐Roussel et al., 2016).

## 11 THE “SHEDDING HYPOTHESIS”

A possible limitation to reptile eDNA studies is that morphological differences in the integument result in different rates of DNA shedding. This has been dubbed the “Shedding Hypothesis” (Adams, Hoekstra, et al., 2019). An animal with a hard or keratinized outer layer (e.g., reptiles) might shed less eDNA than an animal with semi‐permeable skin (e.g., amphibians) (Adams, Hoekstra, et al., 2019; Andruszkiewicz Allan et al., 2020). While eDNA is also shed through feces, urine, saliva, gametes, and physical remains, it is hypothesized that limited eDNA shedding from a keratinized exterior may reduce the detectability of reptiles from environmental samples (Adams, Hoekstra, et al., 2019; Lacoursière‐Roussel et al., 2016; Raemy & Ursenbacher, 2018). Lacoursière‐Roussel et al. (2016) designed two primer pairs to detect reptiles and two primer pairs to detect amphibians in Canadian freshwater systems, and the read abundance was overwhelmingly amphibian (>95%). It is unclear whether these differences were due to differences in integument, abundance, primer design, or other factors.

While the Shedding Hypothesis concept has not been thoroughly tested in reptiles, it has been shown that hard‐shelled organisms have lower shedding rates than soft‐bodied organisms (Andruszkiewicz Allan et al., 2020). Exploring the rates of DNA shedding in reptiles compared to other taxa will allow researchers to better interpret eDNA results, especially when attempting to use universal primers for DNA metabarcoding.

## 12 METHODOLOGICAL CONSIDERATIONS WHEN WORKING WITH eDNA AND REPTILES

PCR inhibition can confound the detection of eDNA by increasing false‐negatives and reducing detection sensitivity, and is common in ecological eDNA studies (Jane et al., 2015). Some sampling environments are more prone to inhibition, including soil, lotic systems, and sediment heavy lentic waters (Jane et al., 2015)—all environments inhabited by reptiles. Inhibition can be circumvented in a number of ways, such as by using specific buffers, inhibition removal kits, diluting samples, or internal positive controls (Adams, Hoekstra, et al., 2019; Jane et al., 2015) (Table S1 in Appendix S1).

**FIGURE 3:**
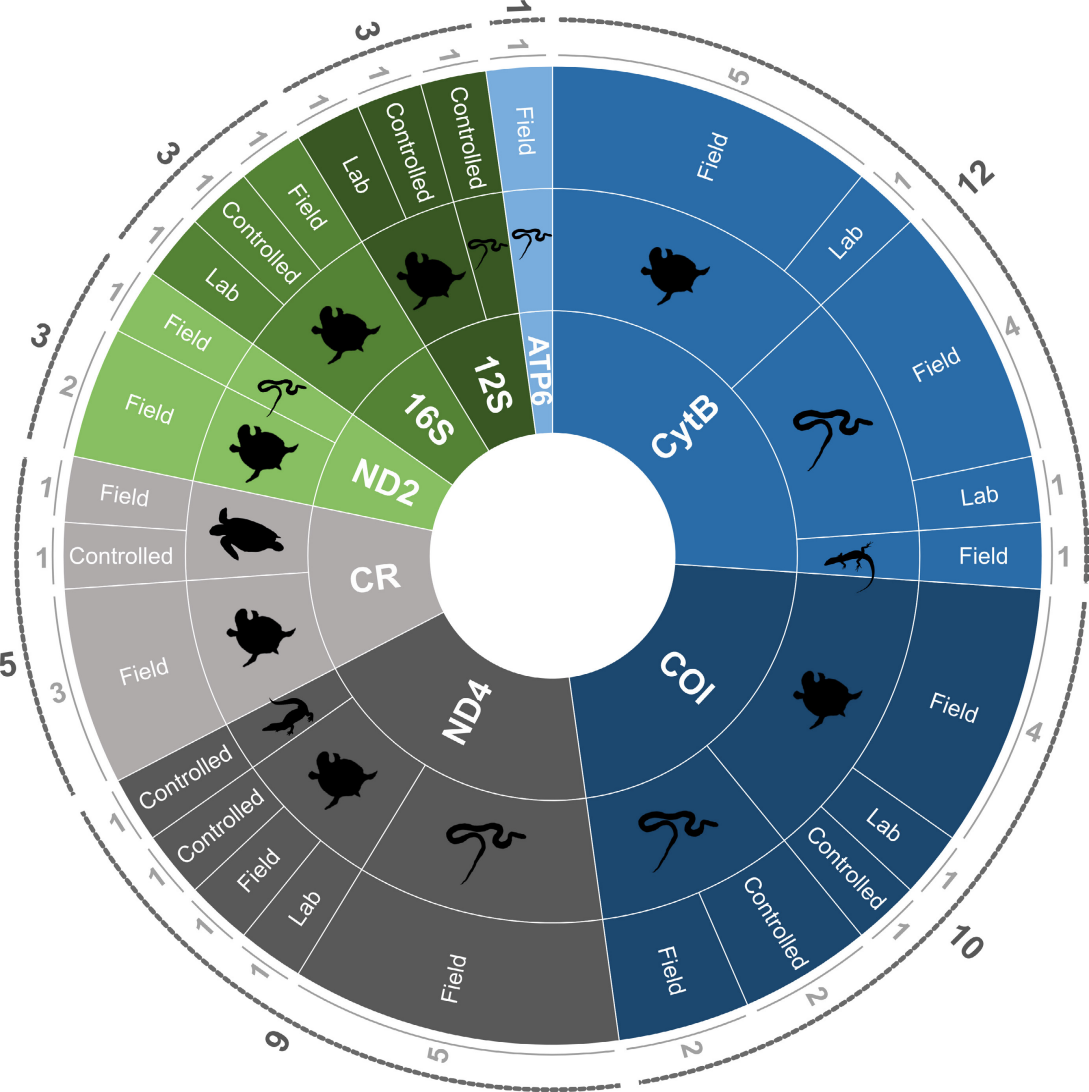
Molecular markers used in reptile species‐specific eDNA studies (inner ring) (CR, control region). Reptile group for each molecular marker is indicated in the middle ring. The outer ring indicates the level of amplification achieved for each reptile group within each molecular marker: Field = studies which successfully amplified the target species under field conditions; Controlled = includes aquaria, terrariums or man‐made enclosures; Lab = target species amplification was achieved in laboratory settings. The numbers on the outer edge indicated the number of studies for each molecular marker, reptile group, and amplification level

Possibly the most important aspect of an eDNA study is the primer‐probe design and validation (Freeland, 2017; Pereira et al., 2019; Wilcox et al., 2013), as primer sensitivity and specificity can impact eDNA amplification. Reptile eDNA studies have used a variety of molecular markers to varying degrees of success, including COI, CytB, ND4, ND2, 12S, 16S, ATP6, control region (Figure 3; Table S1 in Appendix S1). Assays are often developed using available sequences in databases such as GenBank and the Barcode of Life Data Systems (BOLD), and regions may be missing for certain species or taxa (Freeland, 2017). For example, in a metabarcoding study comparing five different genes of reptiles and amphibians, COI and CytB sequence information was available for 31 of the 34 species, whereas sequences for 12S, 16S, 18S were less available (26, 25, and 10 species, respectively) (Lacoursière‐Roussel et al., 2016).

The primary goal of many studies using eDNA is to incorporate the method as a reliable ecological tool into species detection and monitoring. However, the lack of a standardized approach to primer optimization, validation, and reporting has resulted in uncertainty of assay performance, and an inability to directly compare and interpret results across eDNA laboratories, studies, and projects (Agersnap et al., 2017; Klymus et al., 2019; Lesperance et al., 2021; Thalinger et al., 2021; Xia et al., 2021). Standardized methods and reporting metrics are especially important when working with taxa considered difficult to study using eDNA, such as reptiles. eDNA detection difficulties are believed to come from reptiles having lower DNA shedding rates, often being found in low densities, being relatively sedentary, and occurring in habitats with high levels of inhibition. Hence by ensuring assays are rigorously validated and optimized, better understanding will develop of other factors that may affect the performance and efficiency of eDNA detection (Xia et al., 2021).

Best practices can be established and followed during sample collection, which has been the subject of several eDNA studies and reviews (e.g., Buxton et al., 2021; Goldberg et al., 2016; Hinlo et al., 2017; Kumar et al., 2020; Tarof et al., 2021). eDNA sample design should be specific to the organism being targeted (physiology, biology, behavior), and its habitat. It is possible to standardize methods in the laboratory, especially when validating assays to ensure results are comparable across studies and laboratories. For example, consistent methods to report the limit of detection (LOD) and limit of quantification (LOQ) of eDNA assays using qPCR have recently been established (Klymus et al., 2019; Lesperance et al., 2021). Of the 35 eDNA reptile studies that used qPCR for species‐specific detection, nine determined both the LOD and LOQ and provided a definition and detailed methodology of how each metric were calculated. eDNA studies focusing on a broad range of target organisms have already adopted standardized reporting metrics, as outlined in Klymus et al. (2019) and Lesperance et al. (2021), including a study on the sharp‐tailed snake (*Contia tenuis*) (Matthias et al., 2021). Lam et al. (2022) followed a newly established eDNA assay evaluation scale (Thalinger et al., 2021) to validate their big‐headed turtle (*Platysternon megacephalum*) assay, including reporting LOD and LOQ. We anticipate that most eDNA reptile studies published in the future will follow a standardized approach to report LOD and LOQ, which will allow better understanding of assay performance and direct comparison of primers/assays between laboratories and studies.

## 13 FUTURE DIRECTIONS

Molecular ecology and eDNA research continue to rapidly evolve (Jarman et al., 2018), and emerging technologies may offer more sensitive or faster approaches, or the potential to quantify abundance. For example, droplet digital PCR (ddPCR) is quickly being recognized for its potential in eDNA species‐specific detections (Table 1). ddPCR is more sensitive than qPCR, particularly when dealing with low eDNA concentrations (Doi et al., 2015; Mauvisseau et al., 2019; Nathan et al., 2014), and has also been recognized for minimizing effects on PCR inhibitors (Capo et al., 2019; Harper, Lawson Handley, et al., 2019), and shows potential for quantifying abundance (Doi et al., 2015). ddPCR was used in two reptile studies reviewed here (Hunter et al., 2019; Orzechowski et al., 2019), and due to concerns about low DNA shedding rates in reptiles (leading to low eDNA concentrations), the method has the potential to improve the success of eDNA reptile studies.

Another promising molecular approach is the use of isothermal DNA amplification technology, for example, recombinase polymerase amplification (RPA) is an isothermic DNA amplification alternative to PCR. RPA is recognized for its simplicity in sample preparation, sensitivity, and quick reaction time (10–20 min) at low temperatures (37–45°C) with the ability to amplify 1–10 copies of target DNA (Lobato & O'Sullivan, 2018; Wu et al., 2019). RPA has been used in human medicine, agriculture, and food safety (Li et al., 2020; Lobato & O'sullivan, 2018; Rani et al., 2021; Wu et al., 2019); but has not yet been applied to ecological systems. RPA lateral flow (LF) strip assays also being trialed for testing in the field (Li et al., 2020; Rani et al., 2021; Wu et al., 2020), which would offer many benefits when rapid management decisions are needed (e.g., for rare or invasive species).

Nanopore DNA sequencing is gaining attention for its potential use with eDNA (Egeter et al., 2022; Truelove et al., 2019). While traditional DNA sequencing must complete the sequence run before providing data, nanopore sequencing is produced in real time avoiding PCR bias (Johnson et al., 2017). Of particular interest is Oxford Nanopore MinION (Oxford Nanopore Technologies), a light weight (90–450 g), low cost portable DNA sequencing platform that provides rapid real‐time results (Pomerantz et al., 2018) and can be used outside of traditional laboratory settings. For example, Pomerantz et al. (2018) used the MinION and the miniPCR to successfully identify endemic reptile species via DNA barcoding in a global biodiversity hotspot in the Ecuadorian Choco rainforest. This was achieved with high accuracy (>99%) in under 24 h under challenging field conditions (e.g., inconsistent electricity), illustrating how “mobile laboratories that fit in a single backpack” can be used in a conservation context in developing countries (Pomerantz et al., 2018). The MinION has carried over into the field of eDNA (Ames et al., 2021; Egeter et al., 2022; Truelove et al., 2019); for example, eDNA in seawater was sequenced, and annotated results for white sharks (*Carcharodon carcharias*) were available in 48 h, which is a substantial reduction in typical eDNA turnaround times (Truelove et al., 2019).

Further advances in use of mobile approaches was reported by Doi et al. (2021) who developed an in‐field eDNA detection method using an “ultrarapid mobile PCR platform” (mobile PCR), testing it in rivers in lakes to detect silver carp (*Hypophthalmichthys molitrix*). This method achieved measurement time in 30 min while still maintaining high detection sensitivity (Doi et al., 2021). The mobile PCR and MinION nanopore sequencing allow for rapid eDNA detection, cutting down on the lag between collecting eDNA samples and acquiring final results. One benefit of in‐field detections is quicker management decisions of invasive and threatened species (Doi et al., 2021; Egeter et al., 2022). While these methods have not been trialed with reptile eDNA yet, we expect they will be included in reptile management toolboxes moving forward.

eDNA reptile studies most commonly sample aquatic systems by filtering water through a membrane. Active water filtration is the most widely used approach (Rees et al., 2014), but requires specialized equipment and is time‐consuming, which limits sample size. The volume of water filtered is often restricted due to particulates clogging the membrane, which is especially common in sediment‐heavy lentic systems inhabited by reptiles (such as wetlands, bogs, and lakes) (Kirtane et al., 2019). A recent study demonstrated that eDNA collected passively by submerging filter membranes in marine systems can be as effective as active filtration (Bessey et al., 2021). By eliminating the need to filter water, biological replication can be greatly increased, expanding the range of ecological questions that can be answered using eDNA (Bessey et al., 2021). Another approach to passive eDNA sampling in aquatic systems involves suspending PEDS (Passive Environmental DNA Samplers), containing adsorbent materials such as granular‐activated carbon (Kirtane et al., 2020). This approach may also improve eDNA detection in sediment heavy systems (Kirtane et al., 2020), where reptiles can be commonly found but difficult to survey.

eDNA sampling has shown potential in the field of population genetics (Adams, Knapp, et al., 2019; Sigsgaard et al., 2020), especially for marine macro‐organisms (Dugal et al., 2022; Parsons et al., 2018; Sigsgaard et al., 2016). As the field advances, we anticipate eDNA population genetics may be applied to reptiles, allowing for estimates of genetic variation and demographic trends. While most eDNA population genetics studies thus far have focused on population‐level inferences (e.g. Parsons et al., 2018; Sigsgaard et al., 2016), Dugal et al. (2022) obtained accurate individual‐level haplotypes from eDNA. This was accomplished by estimating levels of genetic diversity in a whale shark (*Rhincodon typus*) population by accurately matching individual haplotypes collected from eDNA seawater to individual haplotypes from tissue samples (Dugal et al., 2022). Some future directions of eDNA population genetics research include: incorporating nuclear DNA approaches (Adams, Knapp, et al., 2019; Sigsgaard et al., 2020), abundance estimates using haplotype diversity and frequency (Dugal et al., 2022), and even gene expression using eRNA (Adams, Knapp, et al., 2019; Cristescu, 2019; Sigsgaard et al., 2020).

## 14 CONCLUSIONS

This review illustrates that eDNA can be used successfully with reptiles, although it is clear that success has been difficult to achieve in the past (Baker et al., 2020; van der Heyde et al., 2021). It is important to note that cases where eDNA has been unsuccessful are less likely to be published (Beng & Corlett, 2020), and there is likely a publication bias toward reptile eDNA studies that have had some level of success. The inclusion of eDNA analysis in the management toolbox for reptiles should lead to relatively rapid improvement in knowledge of their distributions and ecological roles. As there are limitations associated with eDNA approaches when working with reptiles, pairing eDNA with traditional sampling and/or site occupancy modeling likely to be an effective way of incorporating eDNA into current monitoring approaches to capture the benefits of this technology. Assay validation and reporting should follow a standardized approach, and eDNA sampling strategies should specifically target a species’ microhabitat use and life cycle (Adams, Hoekstra, et al., 2019). Further, sampling strategies should highly consider a species biology and behavior. To date, reptiles with an aquatic life stage have benefited the most from eDNA approaches; for example, species‐specific eDNA approaches have been used effectively to detect turtles (Table S1 in Appendix S1), illustrating the potential of eDNA for studying one of the most threatened vertebrate groups in the world. Conversely, sampling the eDNA of terrestrial reptiles has not been extensively explored, and eDNA is not being utilized in areas of high reptile species richness. As 20% of reptiles are threatened with extinction, and further 20% are data deficient (Bland & Böhm, 2016), there are many opportunities for application of eDNA (both species‐specific monitoring, and DNA metabarcoding for biodiversity sampling/dietary analysis) in reptile ecology.

## AUTHOR CONTRIBUTIONS

**Bethany Frances Nordstrom:** Conceptualization (lead); Formal analysis (lead); Investigation (lead); Methodology (lead); Visualization (lead); Writing – original draft (lead); Writing – review & editing (equal). **Nicola Mitchell:** Supervision (equal); Writing – original draft (supporting); Writing – review & editing (equal). **Margaret Byrne:** Supervision (equal); Writing – original draft (supporting); Writing – review & editing (equal). **Simon Jarman:** Conceptualization (supporting); Supervision (equal); Writing – original draft (supporting); Writing – review & editing (equal).

## CONFLICT OF INTEREST

The authors declare no conflicts of interest.

## Supporting information

AppendixS1

## Data Availability

There was no new data created or analyzed for this manuscript.
