# Supplementary material for: A review of applications of environmental DNA for reptile conservation and management
Source: Ecol Evol. 2022 Jun 5;12(6):e8995. doi: 10.1002/ece3.8995 (PMC9168342; doi:10.1002/ece3.8995)
Supplement: Supplementary file 1 — AppendixS1 [file ECE3-12-e8995-s001.docx]

**Supporting Information - Appendix 1:**

**Panel S1. Literature review:**

To compare the use of eDNA across the five main vertebrate groups (fish, mammals, birds, amphibians, and reptiles), literature searches were conducted in Scopus in March 2022. The searches were limited to titles, abstracts, and keywords that contained the terms “environmental DNA” OR “eDNA” OR “DNA metabarcoding” and terms specific to each of the taxonomic groups, providing a proxy comparison between the five groups. For mammals, reptiles, and amphibians, taxa specific search terms included orders within the taxonomic group, and common grouping terms (e.g. the reptilian order testudines was included in the search, along with common grouping terms: turtle and tortoise). In contrast, due the large number of fish and bird orders, these groups were searched by class (fish) and sub-order (birds) along with common grouping terms. Note: the search results for fish, birds, mammals, and amphibians were not refined further than this, as the intention was to simply compare search results between the five main vertebrate groups.

The main aim of the review was to examine the utility of eDNA in reptile ecology, conservation, and management, thus the results from the literature search identifying publications focused on eDNA and reptiles were examined further. The Scopus search yielded 87 results, and a secondary search was conducted in Google Scholar for any reptile eDNA studies that may have been missed in Scopus. These were then reduced to 55 peer reviewed articles after manually selecting those that met criteria of an eDNA study with a primary focus on reptile ecology, conservation, or management. Studies that did not have a main focus on reptiles were not included. For example, if a study examining the diet of a predator using DNA metabarcoding of scat found a presence of a reptile, that study was excluded as it was not primarily focused on reptile ecology.

| **Group:** | **Constant Search Terms:** | **Taxa Specific Search Terms:** | **Results:** |
| --- | --- | --- | --- |
| *Fish* | (“environmental DNA” OR “eDNA” OR “DNA metabarcoding”)  AND | (“fish” OR “fishes” OR “fishery” OR “fisheries” OR “myxini” OR “hagfish” OR “cephalaspidomorphi” OR “lamprey” OR “chonrichthyes” OR “elasmobranchii” OR “elasmobranch” OR “shark” OR “skate” OR “ray” OR “sawfish” OR “chimaera” OR “osteichthyes” OR “coelacanth” OR “gar” OR “sturgeon” OR “tarpon” OR “eel” OR “herring” OR “anchovy” OR “salmon” OR “minnow” OR “pike” OR “tetra” OR “smelt” OR “glaxiid” OR “trout” OR “char” OR “perch” OR “haddock” OR “hake” OR “halibut” OR “sculpin” OR “snapper” OR “remora” OR “cichlid” OR “wrass” OR “barracuda” OR “flounder” OR “cod” OR “piranha”) | **978** |
| *Mammal* | (“environmental DNA” OR “eDNA” OR “DNA metabarcoding”)  AND | (“mammal” OR “mammalia” OR “insectivora” OR “mole” OR “shrew” OR “hedgehog” OR “anteater” OR “sloth” OR “armadillo” OR “pholidota” OR “pangolin” OR “chiroptera” OR “bat” OR “carnivora” OR “wolf” OR “coyote” OR “racoon” OR “bear” OR “panda” OR “lion” OR “tiger” OR “feline” OR “fox” OR “leopard” OR “civet” OR “hyena” OR “mongoose” OR “walrus” OR “seal” OR “pinniped” OR “skunk” OR “badger” OR “weasel” OR “otter” OR “rodentia” OR “rodent” OR “rat” OR “mouse” OR “squirrel” OR “capybara” OR “beaver” OR “porcupine” OR “lagomorph” OR “rabbit” OR “hare” OR “perissodactyla” OR “horse” OR “zebra” OR “rhinoceros” OR “tapir” OR “ungulate” OR “deer” OR “pig” OR “hippopotamus” OR “antelope” OR “giraffe” OR “camel” OR “llama” OR “alpaca” OR “sheep” OR “goat” OR “cetacean” OR “whale” OR “dolphin” OR “porpoise” OR “primate” OR “monkey” OR “chimpanzee” OR “gorilla” OR “gibbon” OR “lemur” OR “orangutan” OR “baboon” OR “elephant” OR “hyracoidea” OR “dermoptera” OR “manatee” OR “dugong” OR “aardvark”) | **630** |
| *Bird* | (“environmental DNA” OR “eDNA” OR “DNA metabarcoding”)  AND | (“bird” OR “avian” OR “palaeognathae” OR “ratite” OR “ostrich” OR “emu” OR “cassowary” OR “rhea” OR “tinamous” OR “neognathae” OR “chicken” OR “duck” OR “geese” OR “swan” OR “pigeon” OR “crane” OR “flamingo” OR “grebe” OR “loon” OR “vulture” OR “hawk” OR “falcon” OR “eagle” OR “owl” OR “parrot” OR “passerine” OR “woodpecker” OR “quail” OR “pheasant” OR “grouse” OR “guineafowl” OR “cuckoo” OR “nightjar” OR “sandpiper” OR “gull” OR “tern” OR “penguin” OR “loon” OR “petrel” OR “shearwater” OR “albatross” OR “cormorant” OR “ibis” OR “pelican” OR “heron” OR “shoebill” OR “kingfisher” OR “bee-eater” OR “stork” OR “finch” OR “sparrow” OR “swallow”) | **146** |
| *Amphibian* | (“environmental DNA” OR “eDNA” OR “DNA metabarcoding”)  AND | (“amphibia” OR “amphibian” OR “frog” OR “toad” OR “tadpole” OR “anura” OR “salamander” OR “urodela” OR “caecilian” OR “gymnophiona” OR “newt”) | **208** |
| *Reptile* | (“environmental DNA” OR “eDNA” OR “DNA metabarcoding”)  AND | (“reptile” OR “reptilia” OR “snake” OR “lizard” OR “squamata” OR “tortoise” OR “turtle” OR “testudines” OR “crocodile” OR “alligator” OR “crocodilia” OR “tuatara” OR “rhynchocephalia” OR “terrapin” OR “skink” OR “caiman”) | **99** |

**Table S1** - Summary of 55 reptile eDNA papers from the Scopus literature search, shown in order of year of publication.

| **Reference** | **Group** | **Significance** | **Sample** | **Approach** | **Environment** | **PCR method** | **Molecular marker** | | **Inhibition addressed** | **Outcome** |
| --- | --- | --- | --- | --- | --- | --- | --- | --- | --- | --- |
| Brown et al. 2014 | 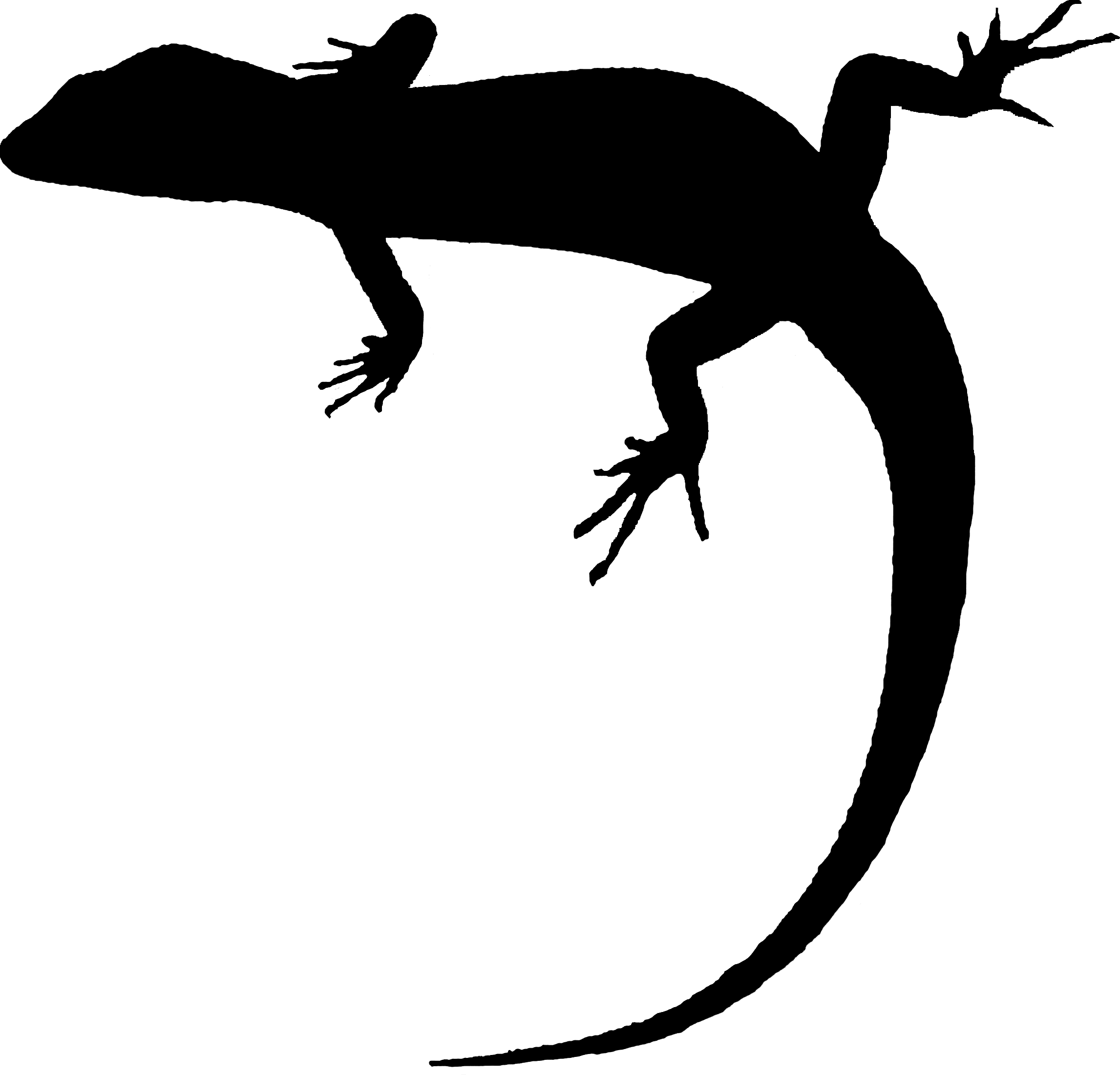 | Threatened  Dietary niche | Faeces | DNA metabar. | --- | --- | COI | N/A | | DNA metabarcoding revealed prey overlap between endemic Telfair’s skink and invasive Asian musk shrew, suggesting potential competition for food resources |
| Kelly et al. 2014 | 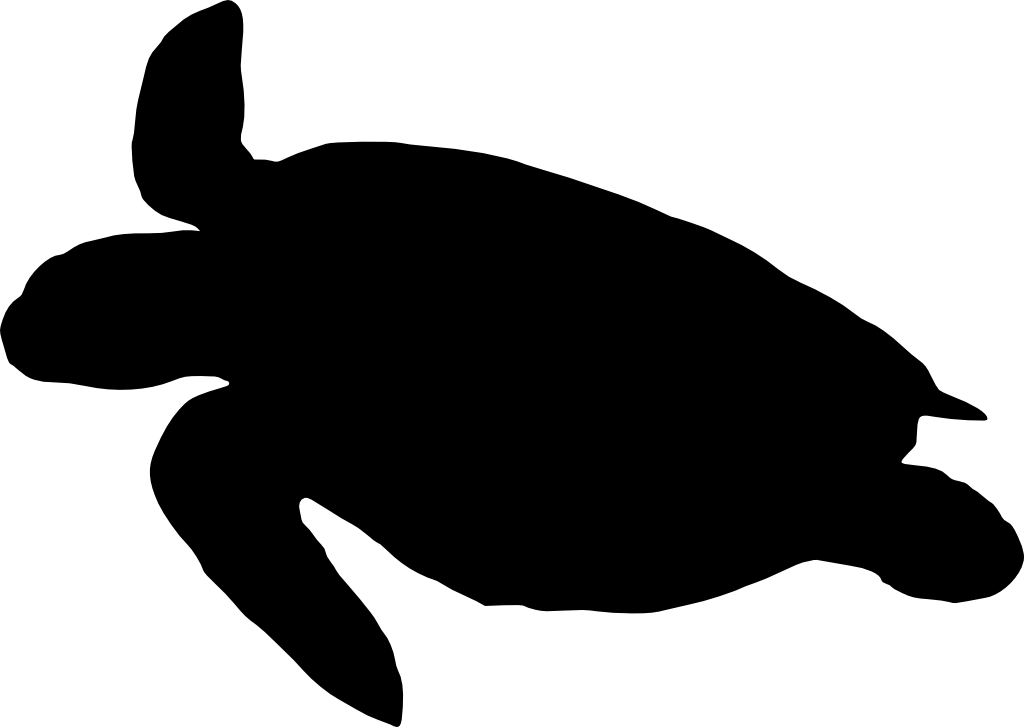 | Threatened | SW  (aquaria) | Species-specific | Cont. | PCR | CR | N/A | | Green sea turtle not identified in DNA metabarcoding (12S), but detected using species-specific primer (CR) |
| Piaggio et al. 2014 | 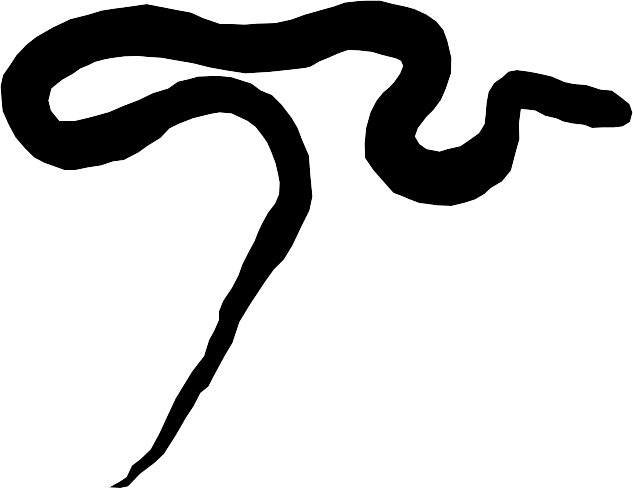 | Invasive | FW  (lentic) | Species-specific | Cont.  Field – U | PCR | CytB | N/A | | Burmese python detected in all water samples, and consistently detectable up to 96 hrs. |
| Davy et al. 2015 | 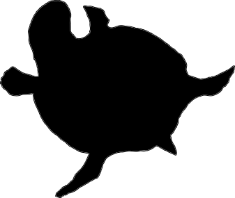 | Threatened  Invasive | FW  (lentic) | Species-specific | Cont. | qPCR / PCR | COI | Dilutions 1:10, 1:20, 1:30 & spiking | | All 9 freshwater turtle species were successfully detected in aquaria (PCR) |
| Hunter et al. 2015 | 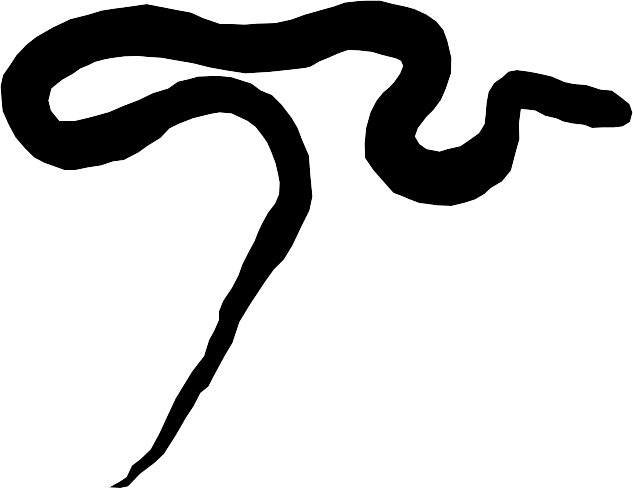 | Invasive | FW  (lentic) | Species-specific | Cont.  Field – K/U | qPCR | ND4  CytB | TaqMan Exogenous Internal Positive Control | | Burmese python eDNA detected in 37 of 63 field samples |
| Kartzinel & Pringle. 2015 | 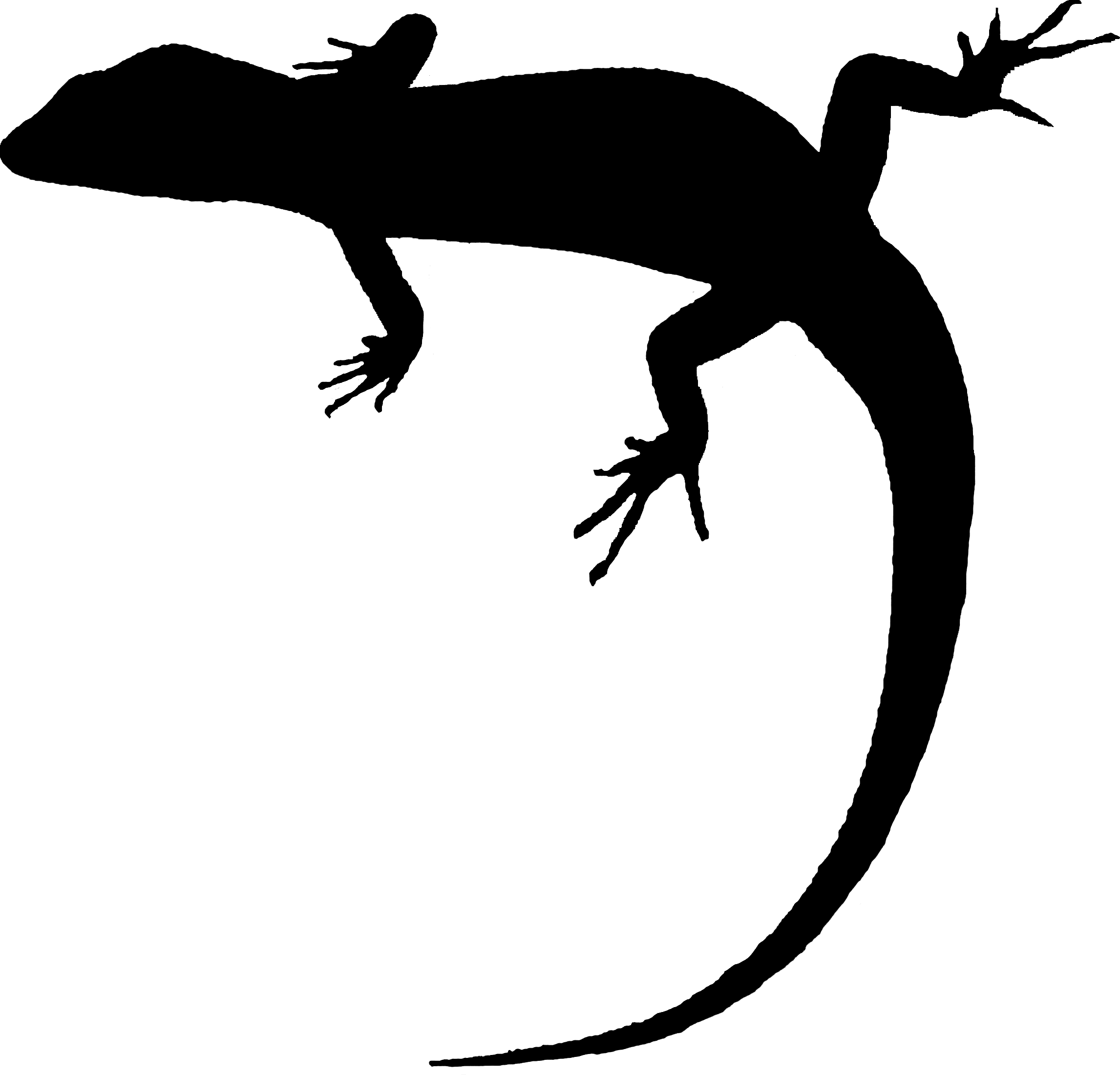 | Dietary niche | Faeces | DNA metabar. | --- | --- | 16S | N/A | | Illumina sequencing revealed three frequent mOTUs (one beetle, cockroach, and ant) and several infrequent mOTUs |
| Clare et al. 2016 | 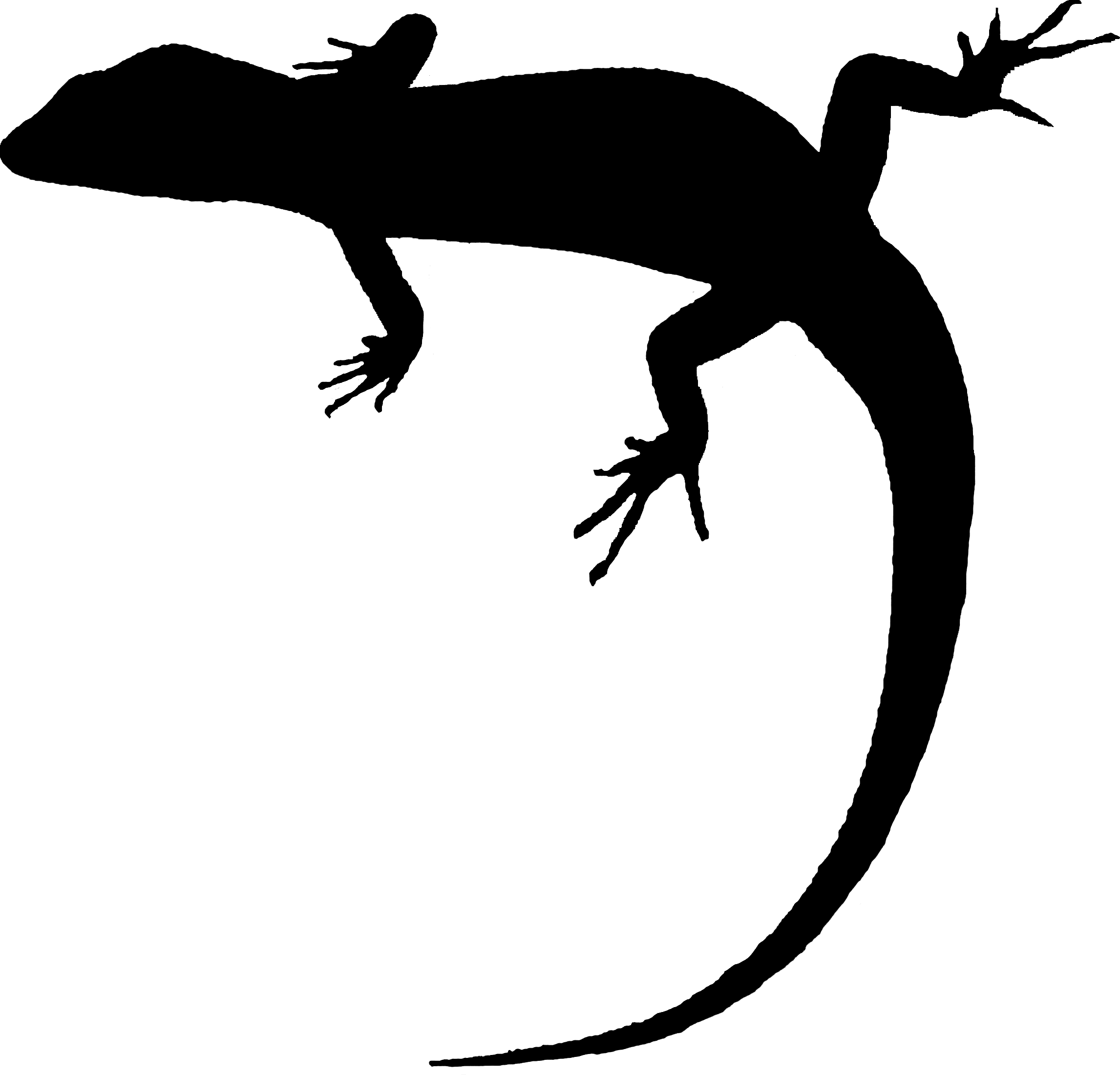 | Dietary niche | Faeces | DNA metabar. | --- | --- | COI | N/A | | Clustering algorithms and filtering thresholds reveal wide variation in niche overlap |
| de Souza et al. 2016 | 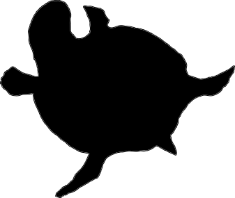 | Threatened | FW  (lotic) | Species-specific | Field – U | qPCR | 16S | N/A | | Flattened Musk Turtle eDNA detected, and season strongly affected eDNA detection probability |
| Koizumi et al. 2016 | 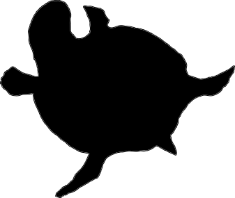 | Invasive  Dietary niche | Faeces | DNA metabar. | --- | --- | Chloroplast | N/A | | Analysis confirmed red-eared sliders feeding on lotus root paddies |
| Lacoursière-Roussel et al. 2016 | 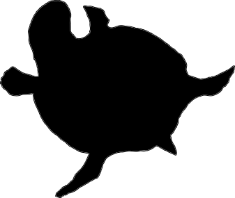 | Threatened | FW  (lentic & lotic) | Species-specific & DNA metbar. | Field – U | qPCR | COI | N/A | | Wood turtle eDNA detection rates provide similar results to standardized visual surveys |
| Feist et al. 2018 | 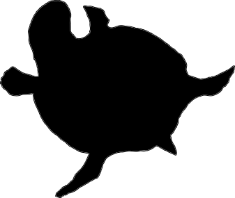 | Threatened | FW  (lentic & lotic) | Species-specific | Cont. | qPCR | CR | TaqMan Environmental Master Mix | | Reliably amplified Alligator snapping turtle eDNA in both lentic & lotic systems without amplifying eDNA from non-target species |
| Kucherenko et al. 2018 | 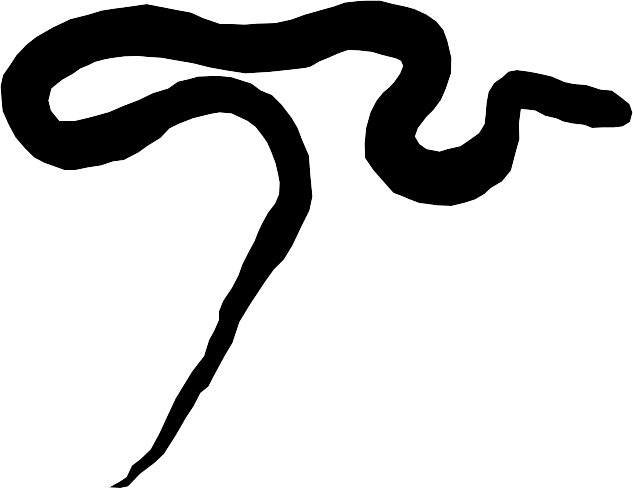 | Invasive | Soil | Species-specific | Cont.  Field – K/U | PCR | COI  12S  CytB | N/A | | eDNA detected 3.5hr after snake had contact with soil and up to 6 days after removal, & detect python eDNA in 66.7% of monitored sites |
| Kundu et al. 2018 | 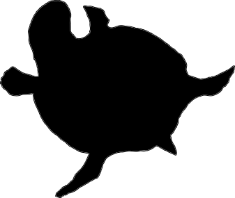 | Threatened | FW  (lentic) | Species-specific | Cont. | PCR | COI | N/A | | Targeted taxa (3 species of FW turtle) were successfully detected from environmental water samples |
| Pinho et al. 2018 | 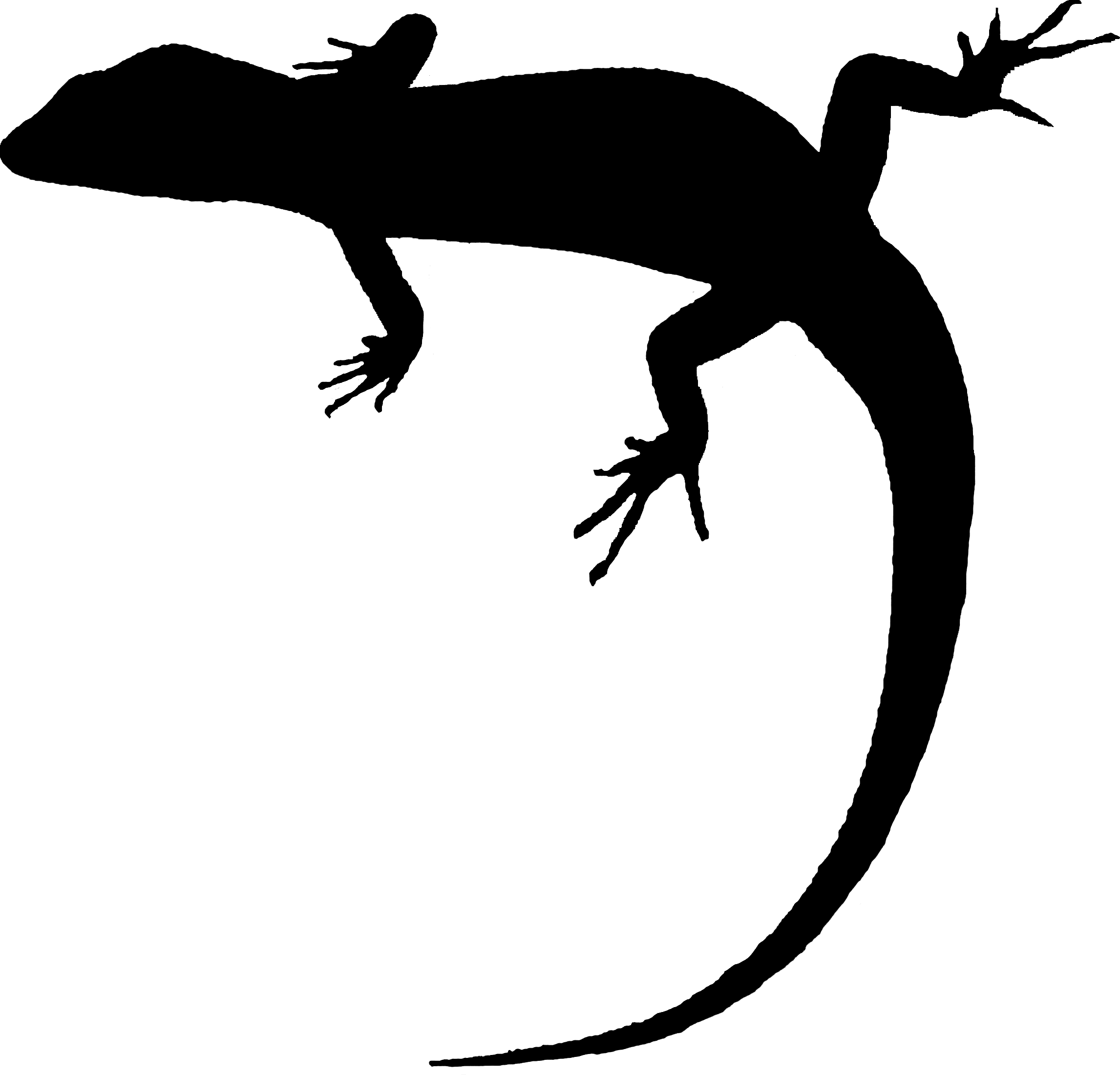 | Threatened  Dietary niche | Faeces | DNA metabar. | --- | --- | trnL  16S  12S | N/A | | Results confirm that the endangered giant wall gecko has a generalist diet, feeding on plants, invertebrates, and vertebrates |
| Raemy & Ursenbacher. 2018 | 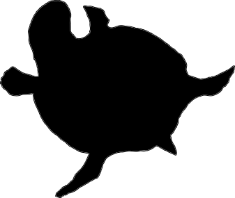 | Threatened | FW  (lentic) | Species-specific | Cont.  Field – K | qPCR | CytB | N/A | | Detection of pond turtle eDNA was higher in artificial ponds with small volumes of water or in the shallow waters of natural ponds |
| Rivera et al. 2018 | 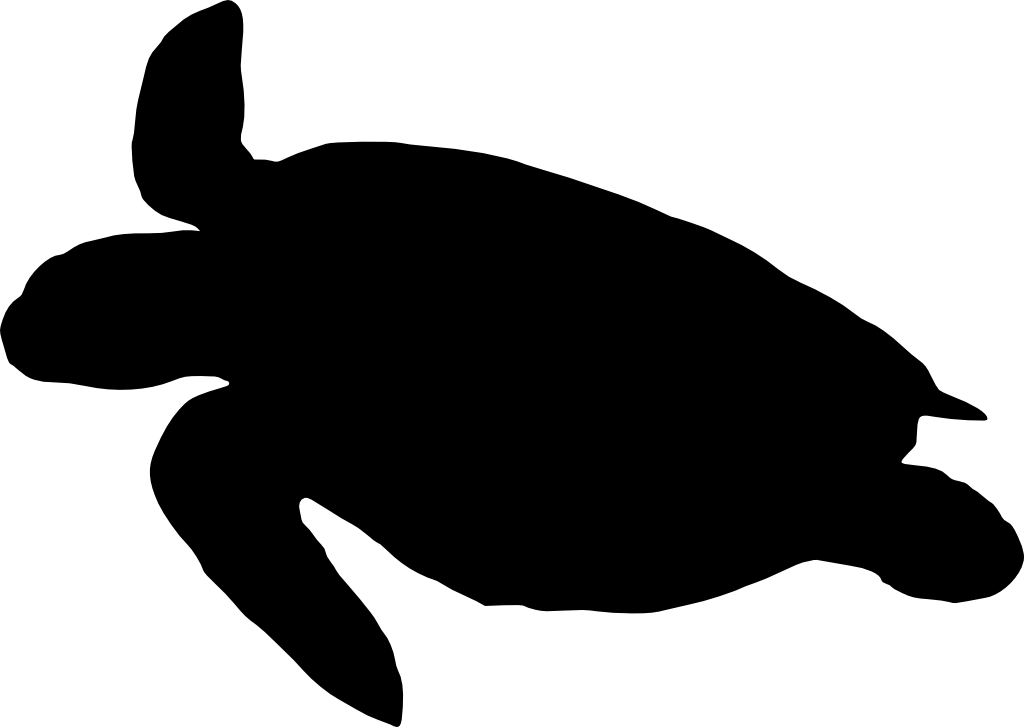 | Threatened  Ecological roles | Epibiotic (Scraping) | DNA metabar. | Field – K | --- | rbcl | N/A | | DNA metabarcoding revealed ability to differentiate turtles based on diatom communities |
| Schumer et al. 2018 | 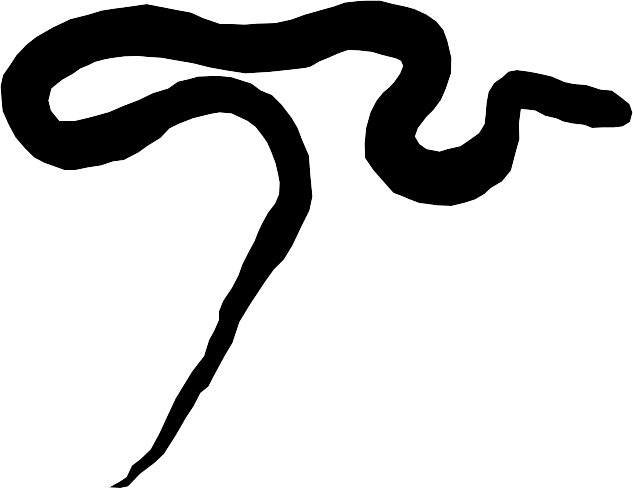 | Threatened | FW  (lotic) | Species-specific | Field – K/U | qPCR | CytB,  ND4 | N/A | | Detected giant gartersnake eDNA in 28/52 locations samples where physical sampling failed |
| Wilson et al. 2018 | 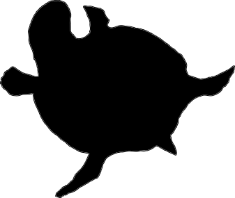 | Threatened | FW  (lotic) | Species-specific | Cont.  Field – K | PCR | CytB | N/A | | Successfully detected Southern river terrapin eDNA from aquariums and field sites |
| Adams et al. 2019 | 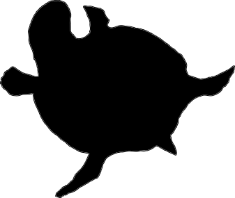 | Cryptic | FW  (lentic) | Species-specific | Cont. | qPCR | COI | Environmental Master Mix 2.0, & ToughMix | | Painted turtle eDNA concentrations were rank-order correlated with density |
| Akre et al. 2019 | 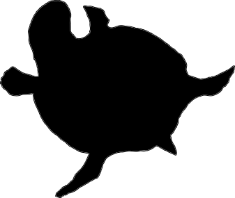 | Threatened | FW  (lotic) | Species-specific | Field – U | qPCR | CR | BSA | | Wood turtle eDNA detected at 76% of sites confirmed by visual surveys, and 3 sites where not detected by visual surveys |
| Hunter et al. 2019 | 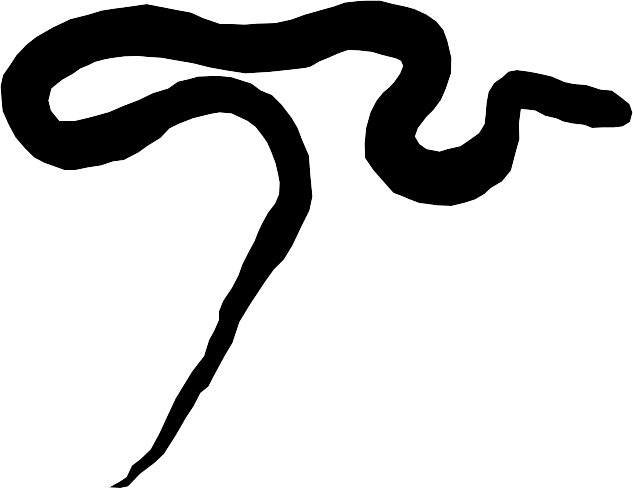 | Invasive | FW  (lentic) | Species-specific | Field – U | qPCR / ddPCR | ND4 | OneStep PCR IRK | | Burmese python eDNA occurrence : 58 – 91%, and detection probability: p = 38 – 70% (potentially reduced by inhibition) |
| Kakuda et al. 2019 | 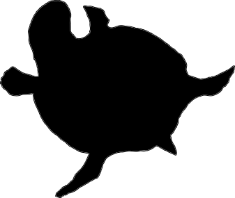 | Invasive | FW  (lentic) | Species-specific | Field – K | qPCR | CytB | Compared Ct shift & spiking | | Red-eared slider detected in 30/100 ponds; & found significant positive correlation between observed number of turtles & eDNA concentration |
| Kirtane et al. 2019 | 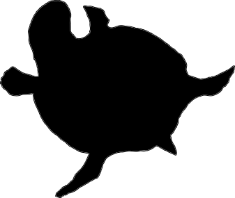 | Threatened | FW  (lentic) | Species-specific | Field – K/U | qPCR | COI | Applied kinetic outlier detection methods  & spiking | | Lower than expected detection rates of bog turtle in contrived samples (54%). Only 2 of 6 non-contrived samples detected eDNA (with 20-75% detection rate) |
| Lam et al. 2019 | 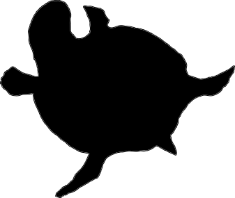 | Threatened  Invasive | FW  (lentic) | Species-specific | Cont. | qPCR | ND4  16S  12S | TaqMan Exogenous Internal Positive Control | | Three assays developed for three species of turtle – 2 worked well (no signs of non-target species amplification), and 1 amplified non-target species DNA. |
| Lopes et al. 2019 | 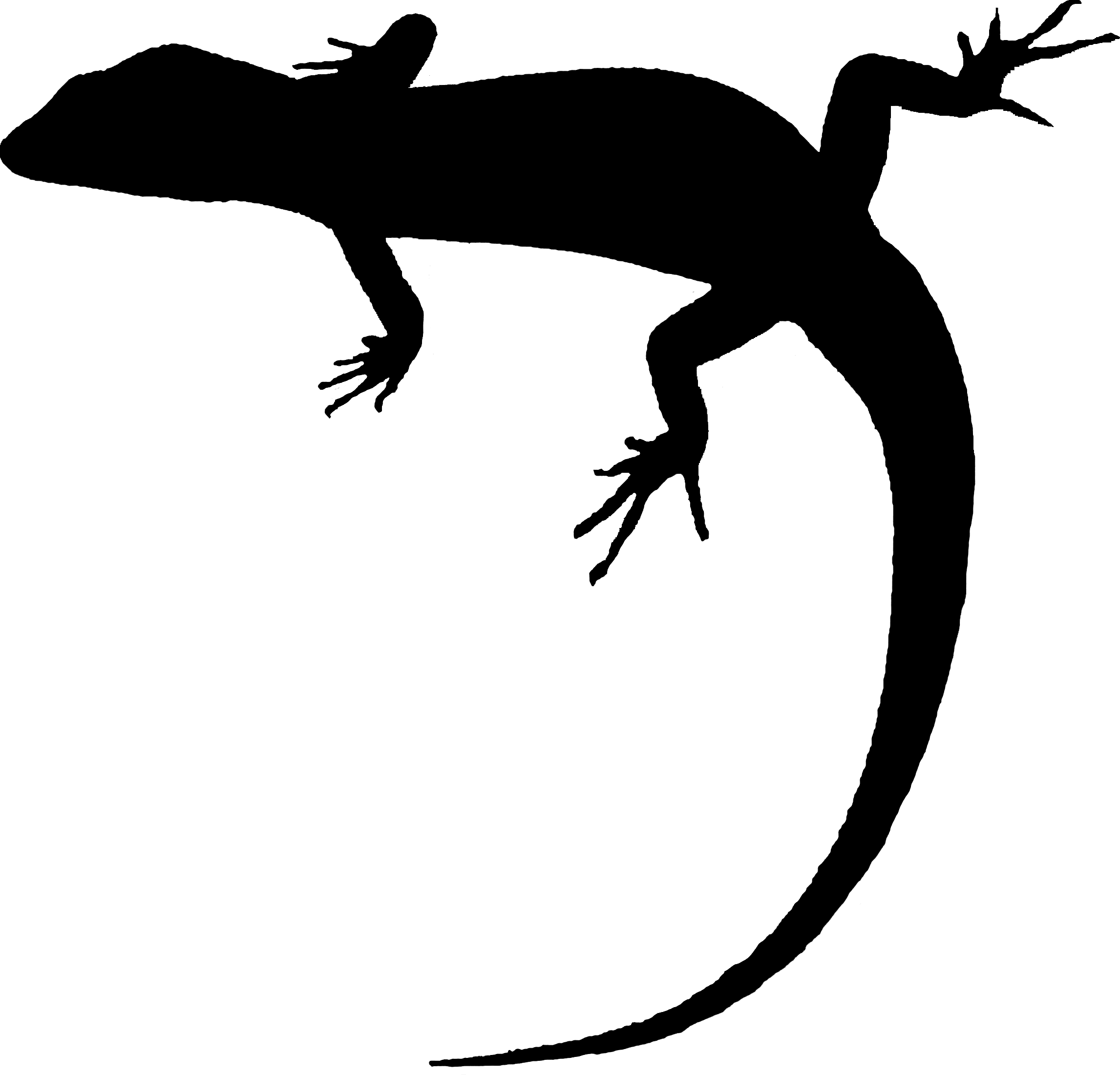 | Threatened  Dietary niche | Faeces | DNA metabar. | --- | --- | 12S | N/A | | Results revealed Endangered giant wall gecko predates on critically endangered raso lark |
| Orzechowski et al. 2019 | 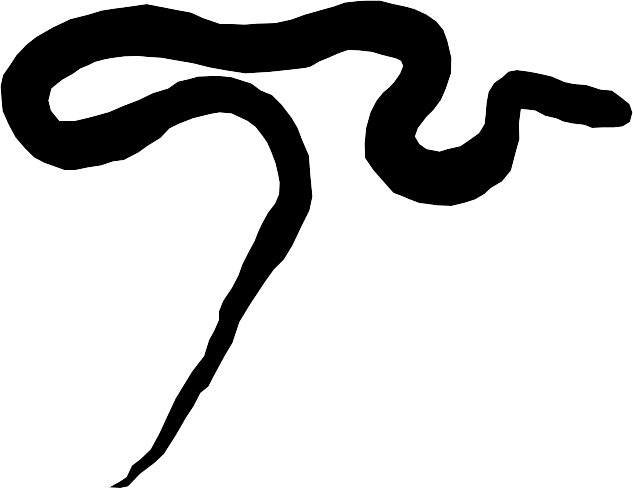 | Invasive | FW  (lentic) | Species-specific | Field – U | ddPCR | ND4 | OneStep PCR IRK | | Detected Burmese python eDNA at 10/15 bird colony sites, and 4/15 control sites, showing higher detection of pythons at wading bird colonies |
| Pereira et al. 2019 | 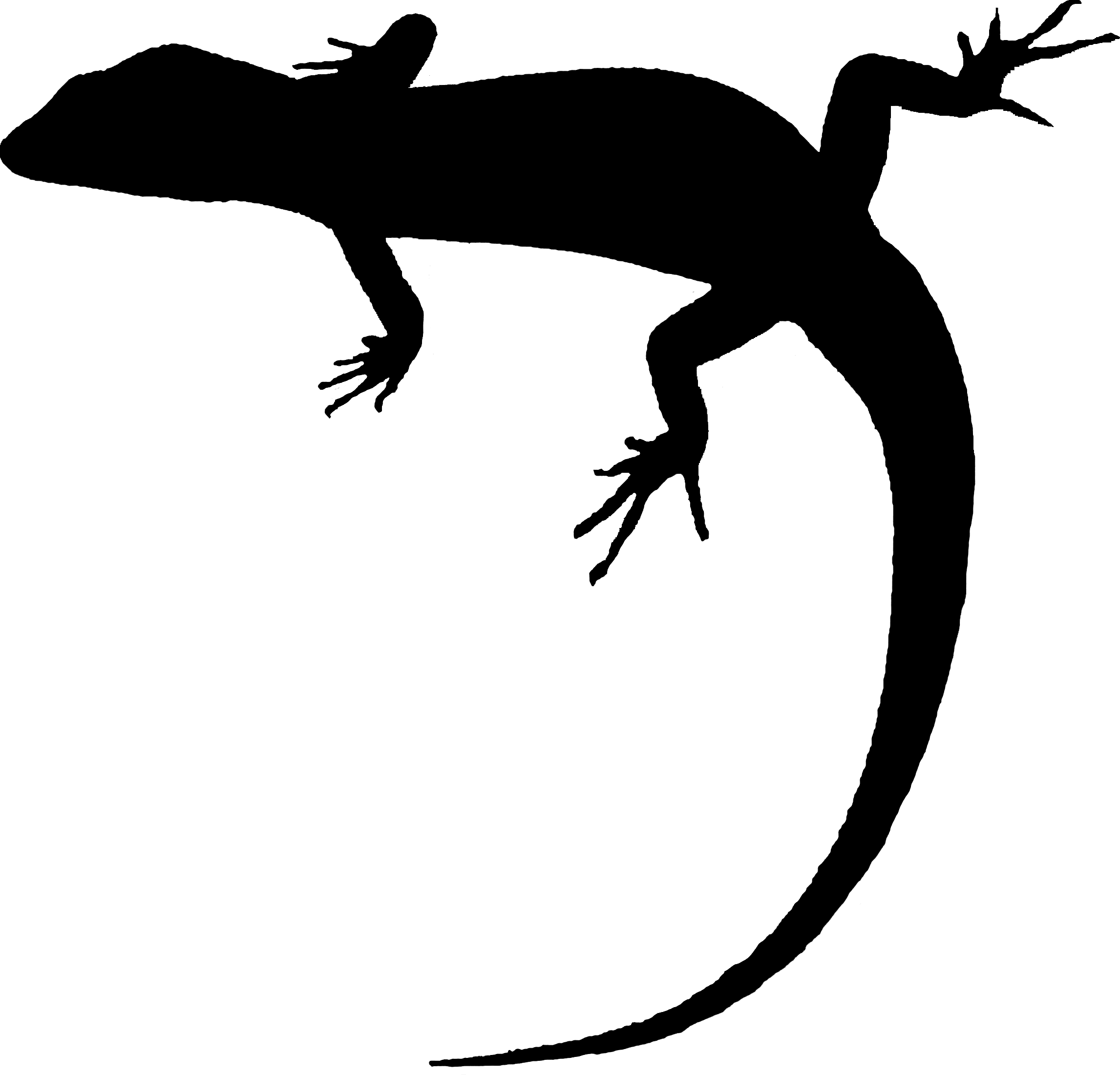 | Dietary niche | Faeces | DNA metabar. | --- | --- | 16S  COI | N/A | | Dietary behaviour was determined for three syntopic lizard species |
| Reinhardt et al. 2019 | 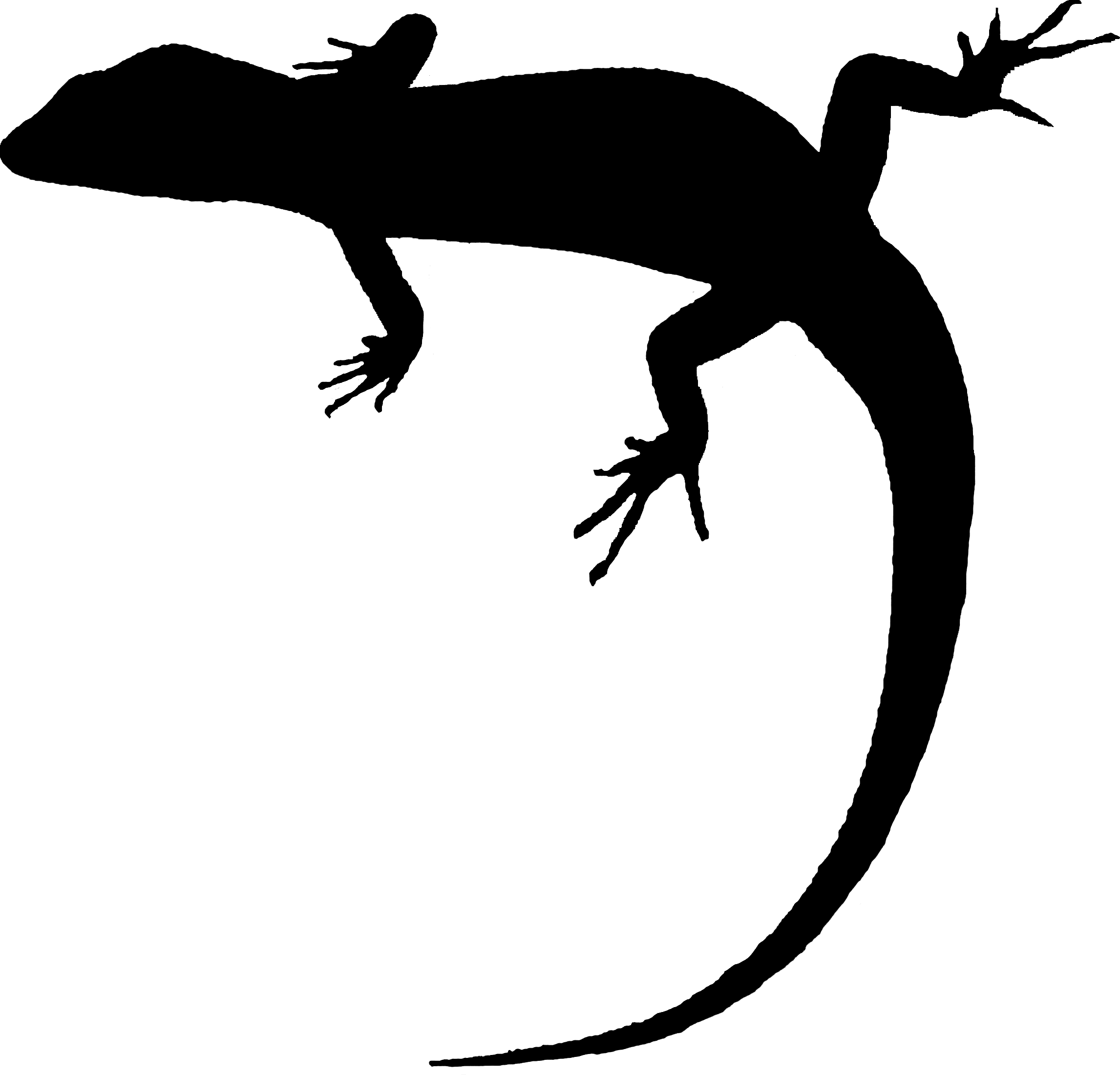 | Threatened | FW  (lotic) | Species-specific | Cont.  Field – U | qPCR | CytB | N/A | | eDNA of Crocodile lizard was detected in all positive controls, and in 4/6 natural sites |
| Rose et al. 2019 | 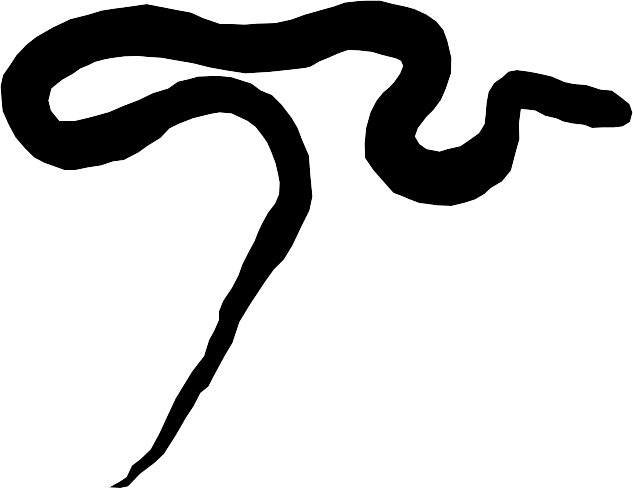 | Invasive | FW  (lentic & lotic) | Species-specific | Field – K/U | qPCR | ND2 | Gene Releaser & TaqMan Exogenous Internal Positive Control | | *Nerodia fasciata* eDNA detected in 3/9 samples, and *N. sipedon* in 5/6 samples |
| Baker et al. 2020 | 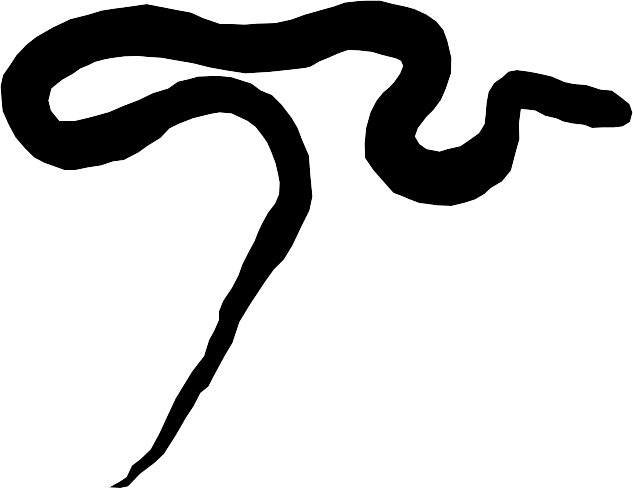 | Threatened  Disease | FW  (lentic) | Species-specific | Field – U | qPCR | COI | Zymo PCR IRK | | Eastern massasauga rattlesnake eDNA detected in 2/100 burrows, even with high densities in close proximity |
| Ducotterd et al. 2020 | 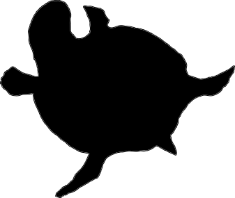 | Threatened  Dietary niche | Faeces | DNA metabar. | --- | --- | rbcL  matK  28S  trnL-trnF  COI | N/A | | Precisely determined omnivorous and opportunistic diet of the European pond turtle, and showed it is not a threat to its environment (and other Red List species) |
| Feng et al. 2020 | 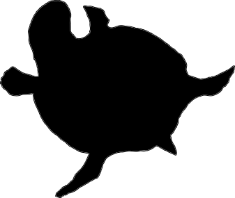 | Threatened | FW  (lentic) | Species-specific | Field – K/U | qPCR | cytb | N/A | | Detected northern map turtle eDNA at known overwintering sites, and at one previously suspected site (confirmed with ROV survey) |
| Gil et al. 2020 | 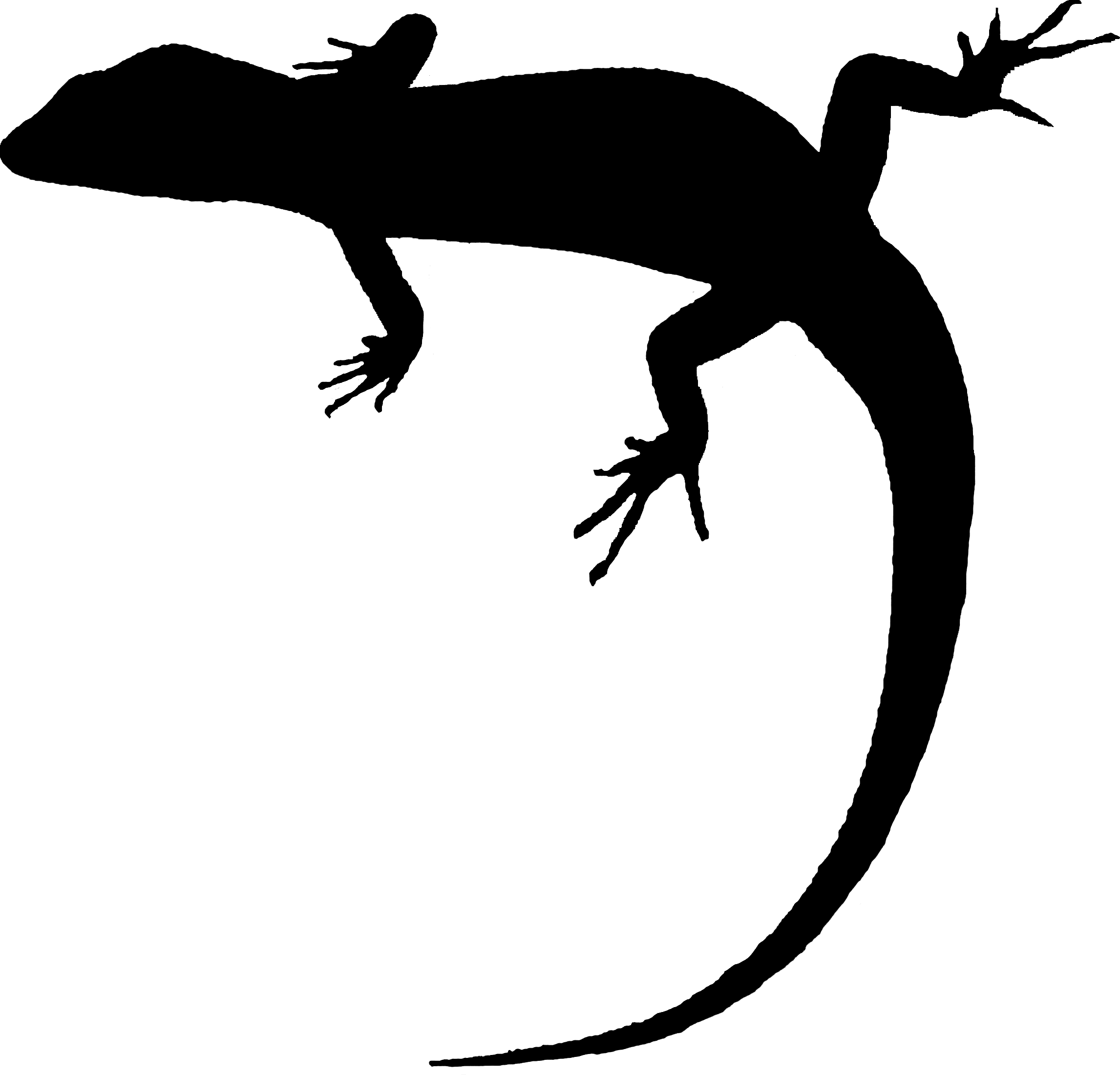 | Threatened  Dietary niche | Faeces | DNA metabar. | --- | --- | 16S  COI  12S  Chloroplast trnl | N/A | | DNA metabarcoding revealed the Selvagens gecko has a more diverse diet than previous morphological identification of faecal pellets |
| Harper et al. 2020 | 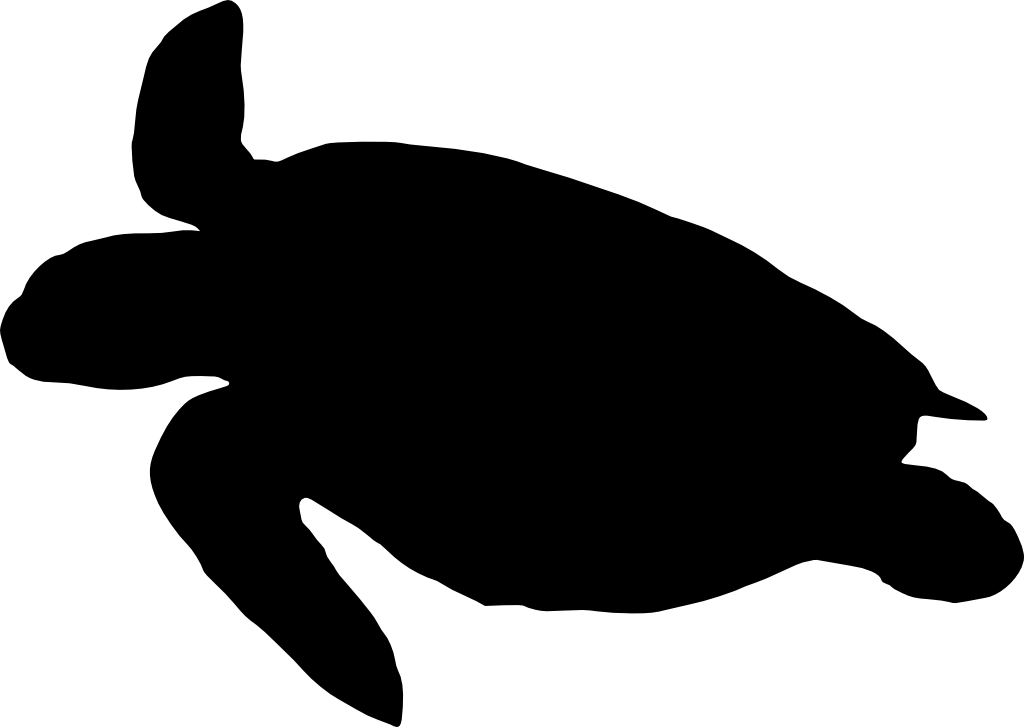 | Threatened | SW | Species-specific | Cont.  Field – K/U | qPCR | d-loop | Bovine Serum Albumin (BSA) | | Detection of Green sea turtle in all aquaria samples, and 7/12 field samples |
| Kessler et al. 2020 | 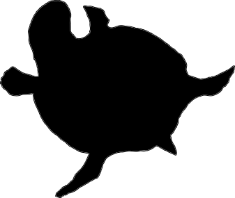 | Threatened | FW  (lotic) | Species-specific | Field – K/U | qPCR | CR | PCR OneStep IRK | | Alligator snapping turtle eDNA detected in 91 site replicates from 19 sites, and eDNA occupancy was impacted by upstream turtle biomass and UV exposure |
| Loeza-Quintana et al. 2020 | 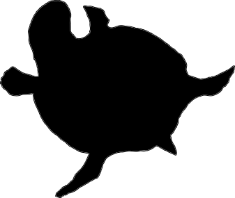 | Threatened | FW  (lentic & lotic) | Species-specific | Field – K/U | qPCR | ND2 | PCR OneStep IRK | | After inhibition removal treatment, Blanding’s turtle eDNA was detected in 2/5 sites (one being an expected negative site) |
| Montoya-Ciriaco et al. 2020 | 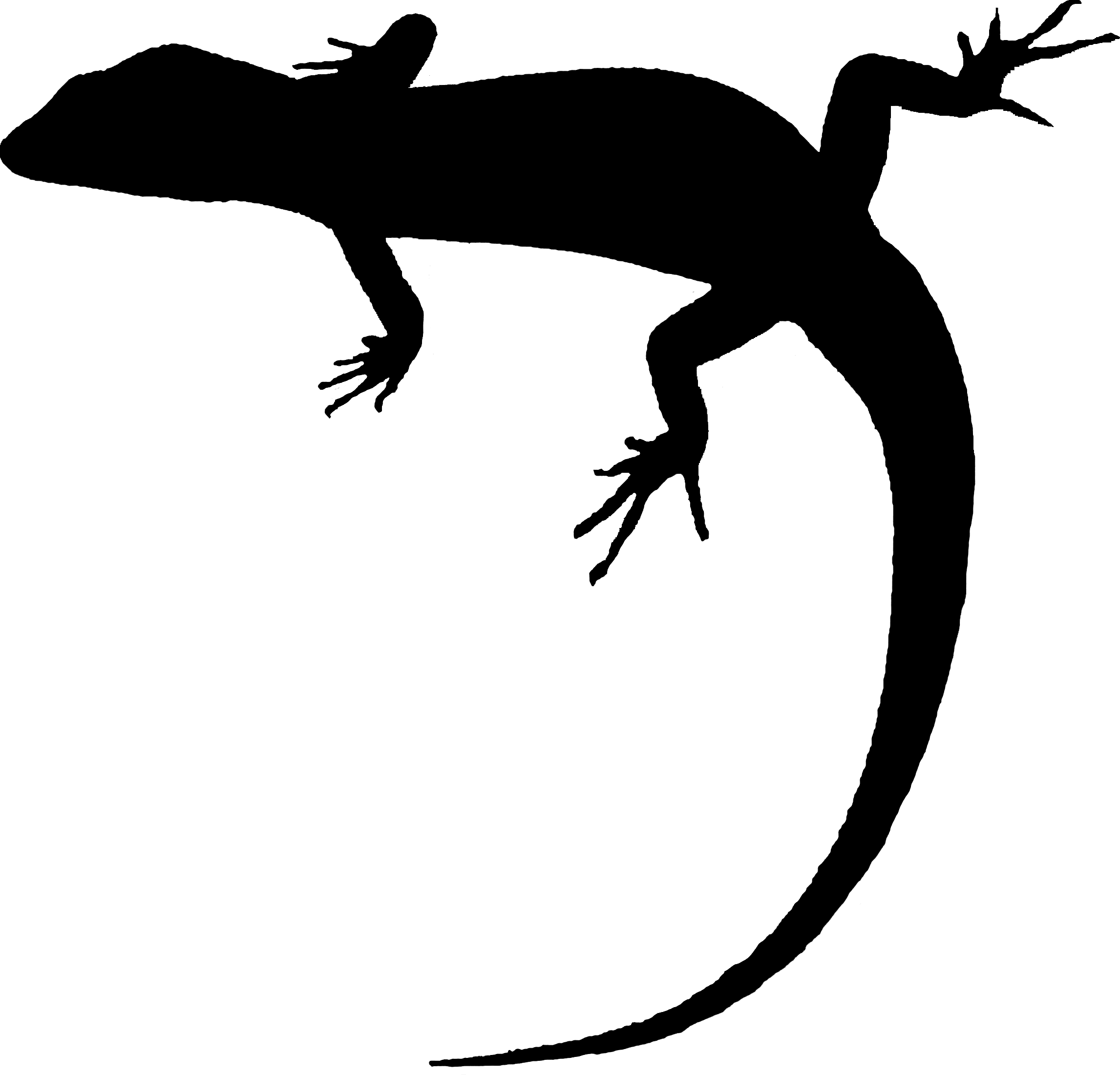 | Microbiome function | Faeces | DNA metabar. | --- | --- | 16S  18S  COI | N/A | | Diversity of diet decreased as altitude increased, and differences in faecal microbiota were revealed at different altitudes |
| Ratsch et al. 2020 | 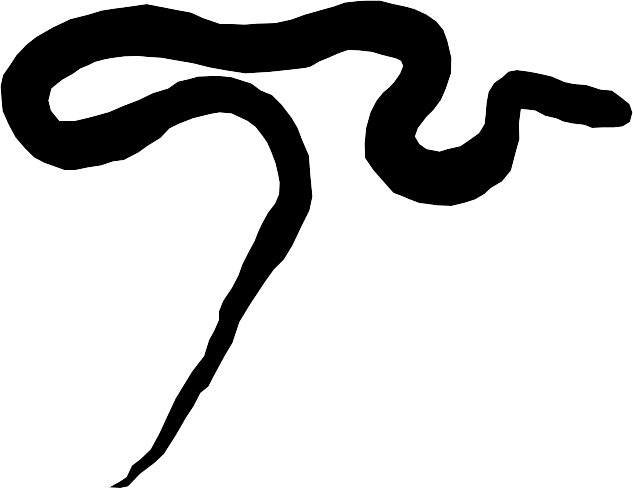 | Threatened | FW, sediment, soil | Species-specific | Field – K/U | qPCR | COI | PVP added to CTAB | | Sediment from one crayfish burrow detected Kirtland’s snake eDNA (out of 380 total samples) |
| Rose et al. 2020 | 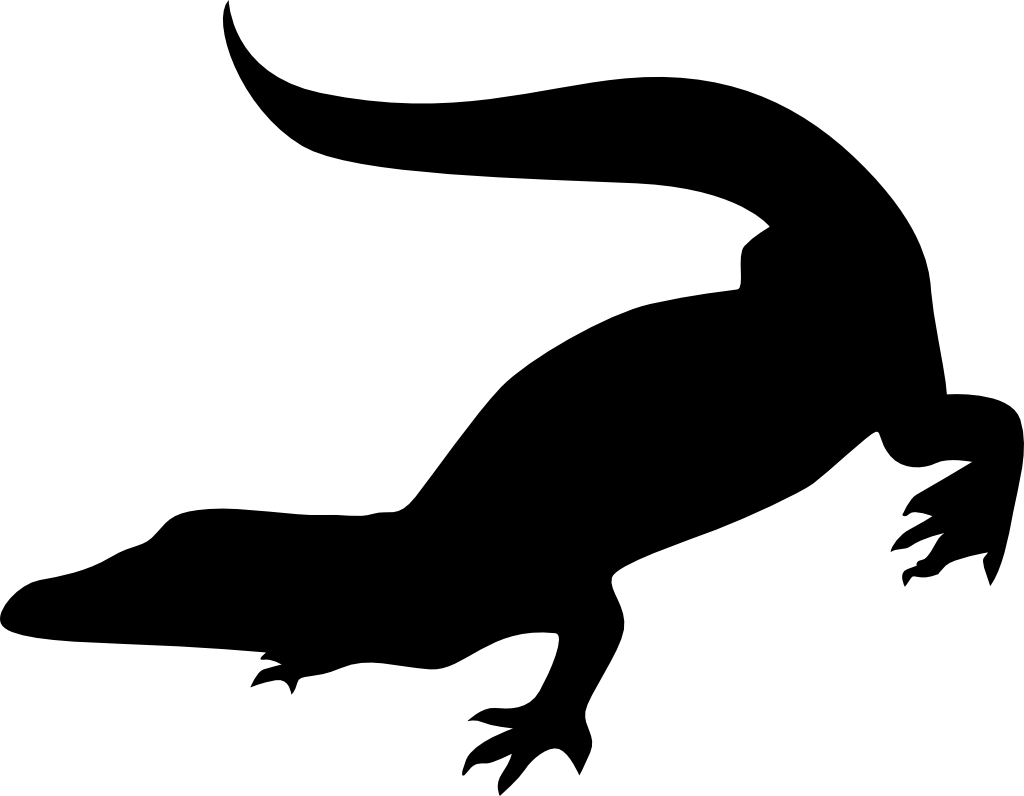 | Wildlife-human interaction | FW | Species-specific | Cont. | qPCR | ND4 | N/A | | Crocodile DNA detected in aquaria ponds within an hour of entry, and 72 hours after removal |
| So et al. 2020 | 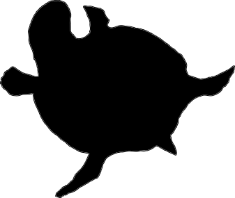 | Threatened  Invasive  Primer specificity | Tissue (in vitro) | Species-specific | --- | PCR / qPCR | COI  ND4  Cytb  16S  12S | N/A | | Results indicated that primer specificity based on the number and position of mismatches can be misleading |
| Anslan et al. 2021 | 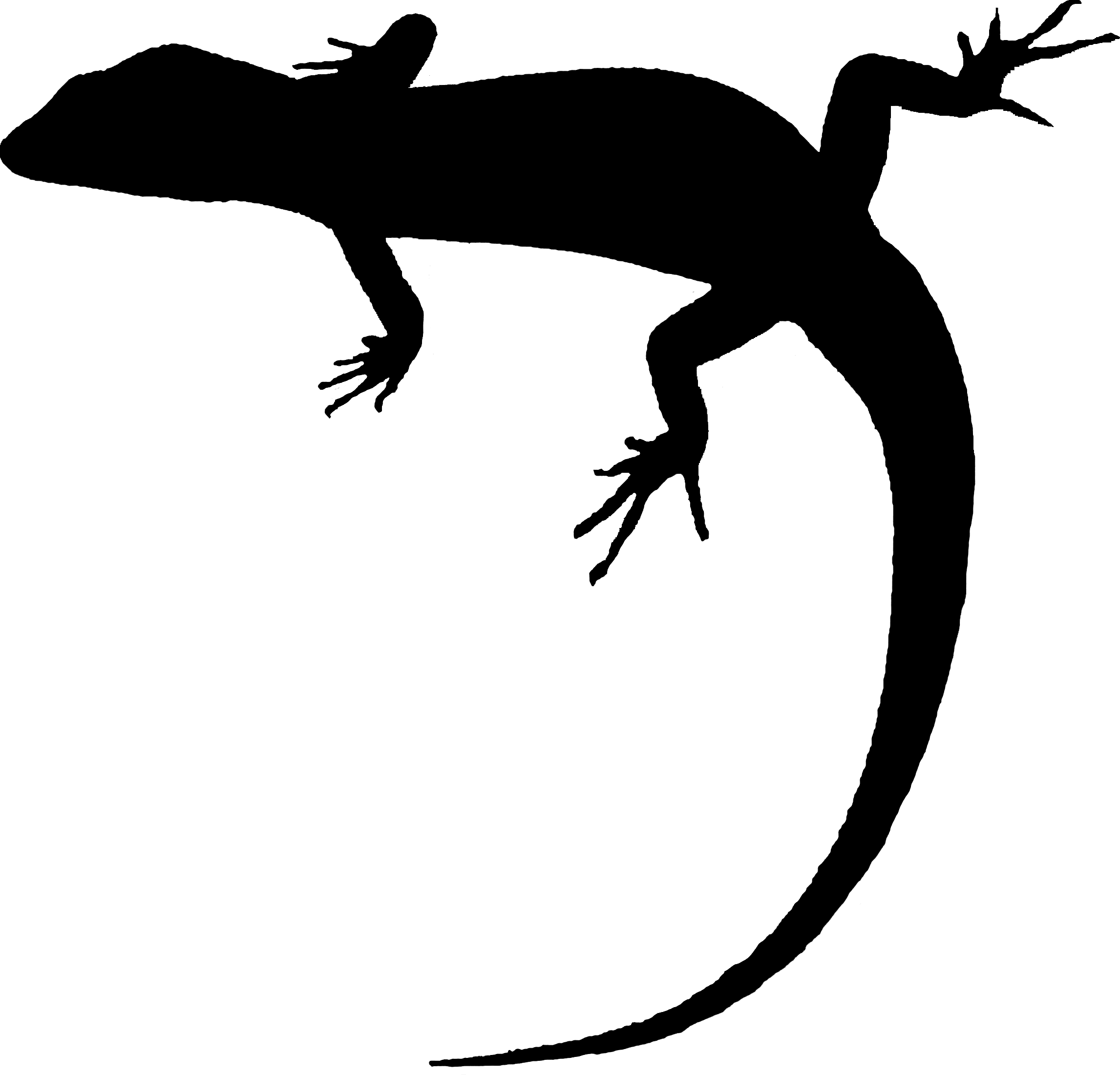 | Dietary niche | Faeces | DNA metabar. | --- | --- | rbcL | N/A | | There was no difference in number of algae species consumed between the two marine iguana subspecies, however, there was low diet overlap, suggesting the subspecies consume different algae species |
| Ducotterd et al. 2021 | 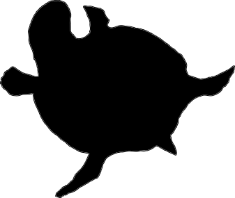 | Threatened  Dietary niche | Faeces | DNA metabar | --- | --- | COI  matK  rbcl  28S  trnL-trnF | N/A | | A novel long DNA metabarcoding method precisely identified diet of European pond turtle in feeding trials and in field settings. |
| Fyson & Bouin-Demers 2021 | 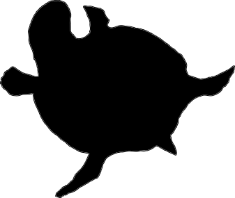 | Threatened | FW  (lentic) | Species-specific | Field – U/K | qPCR | N/A | N/A | | 26 of 89 sites tested positive for Blanding’s turtle DNA. 11 of 23 positive control wetlands amplified Blanding’s turtle DNA. |
| Katz et al. 2021 | 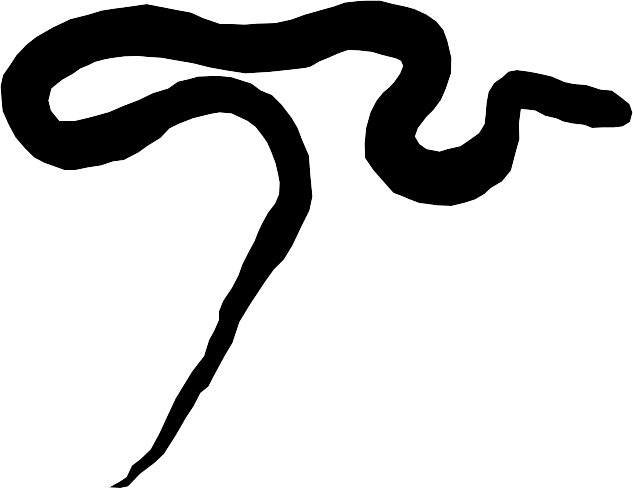 | Threatened | Soil | Species-specific | Cont.  Field - K | qPCR | COI  ATP6 | N/A | | Both assays were capable of detecting Louisiana pinesnake in bedding and soil samples in controlled and field environments |
| Martin et al. 2021 | 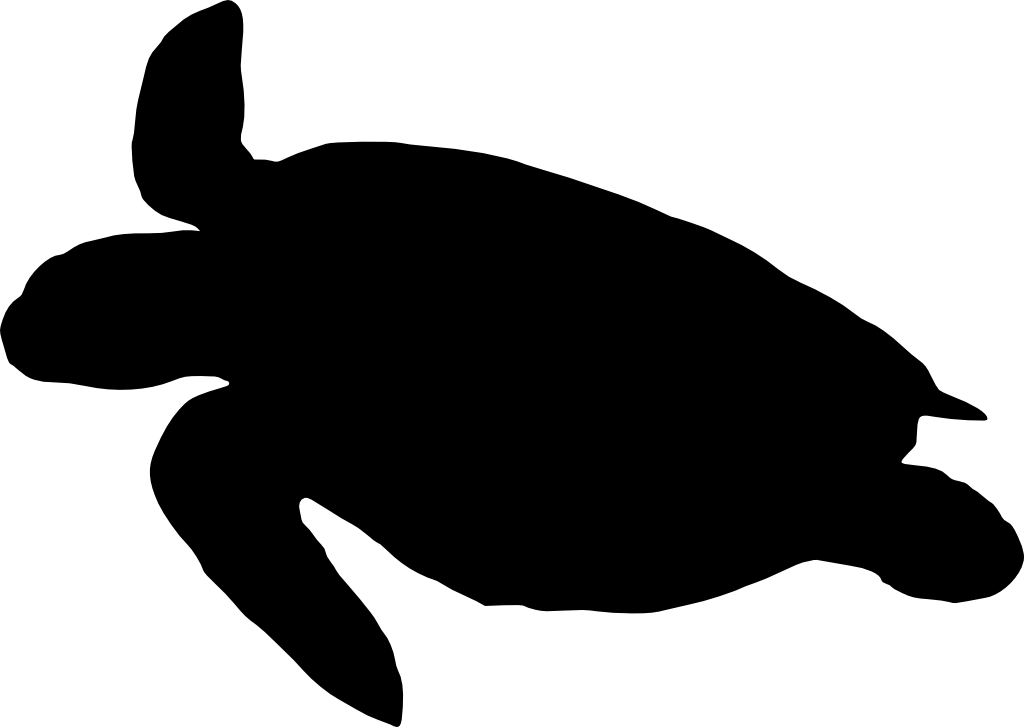 | Threatened  Dietary niche | Gut content | DNA metabar. | --- | --- | 18S | --- | | Visual observation and DNA metabarcoding of stomach contents were complementary to cover loggerhead turtle prey. Dietary DNA detected ctenophores while visual observation did not. |
| Matthias et al. 2021 | 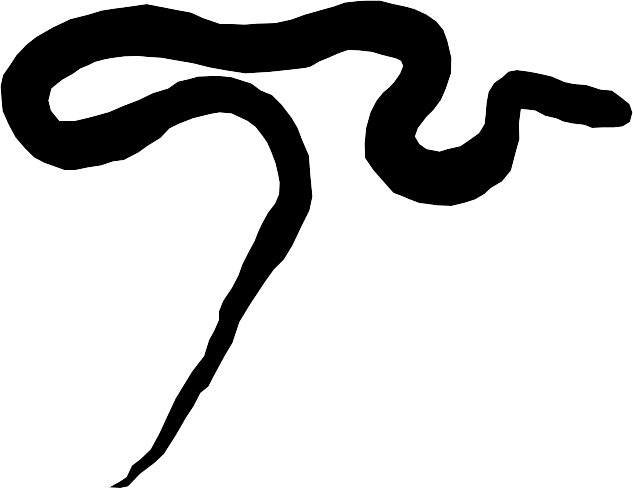 | Threatened | Soil | Species-specific | Field – U/K | qPCR | NADH4 | Zymo OneStep IRK | | The eDNA assay had an overall detection rate of 25% under artificial cover objects, as opposed to a 0.4% visual survey detection rate |
| Rajabizadeh et al. 2021 | 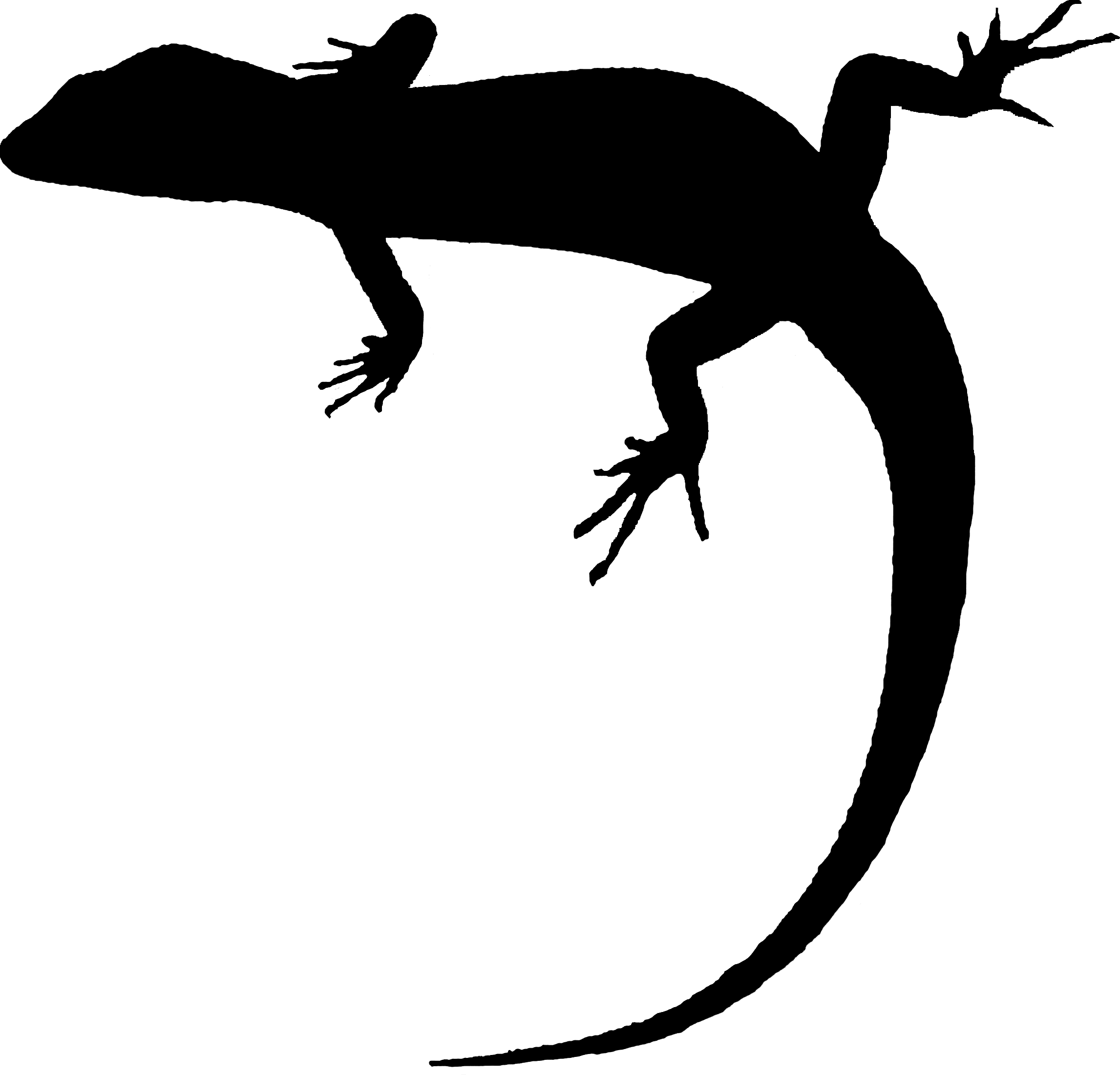 | Dietary niche | Stomach | DNA metabar. | --- | --- | COI | N/A | | DNA metabarcoding revealed Misonne’s spider gecko is a generalist which strongly relies on insects thatmigrate from surrounding areas into the Lut Desert (an extreme desert environment) |
| Siler et al. 2021 | 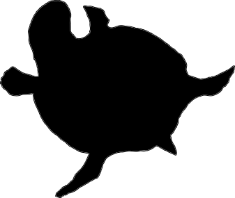 | Threatened | FW  (lentic) | Species-specific | Field – U | qPCR | cytb | PCR OneStep IRK | | Assays detected the presence of the Western Chicken Turtle from a single waterbody (very low detection rate) |
| Tarof et al. 2021 | 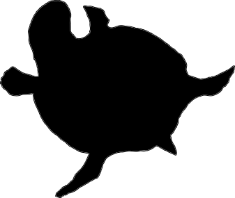 | Threatened | FW  (lentic) | Species-specific | Cont.  Field - K | qPCR | NADH2 | Zymo OneStep IRK | | 8 of 17 wetland sites positive detections of turtles using traditional sampling methods. 50% (n = 4) of these positive control sites amplified Blanding’s turtle DNA |
| West et al. 2021a | 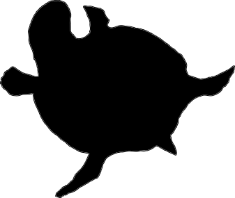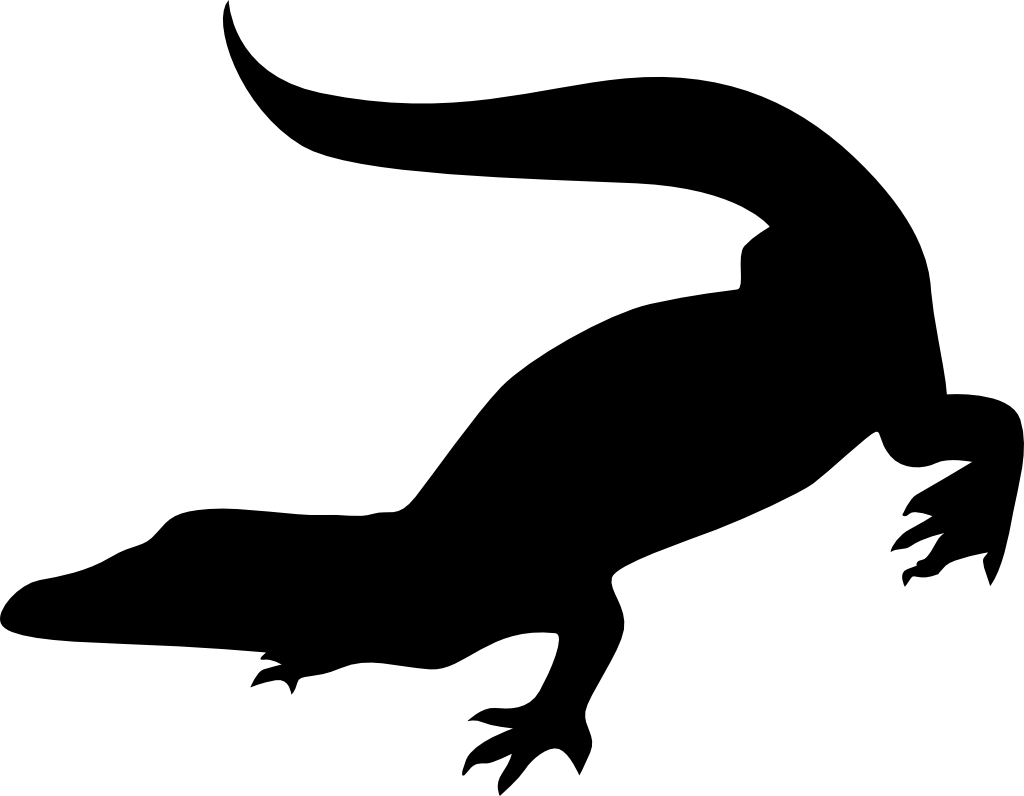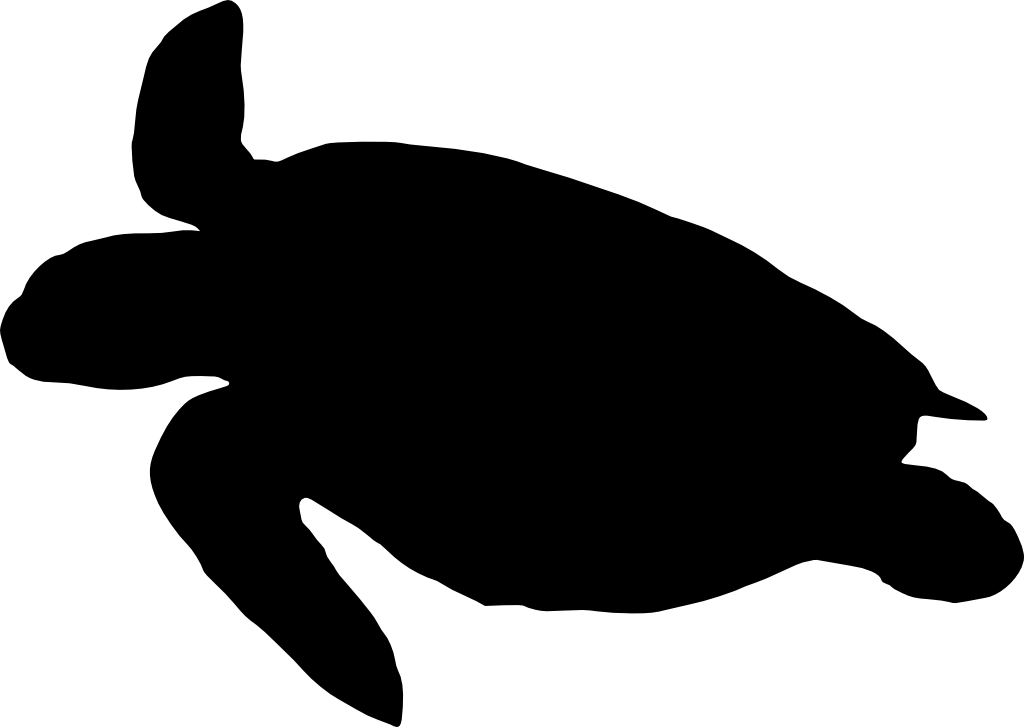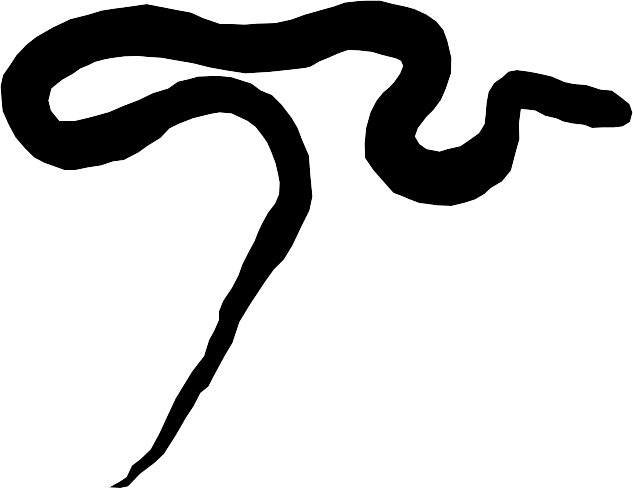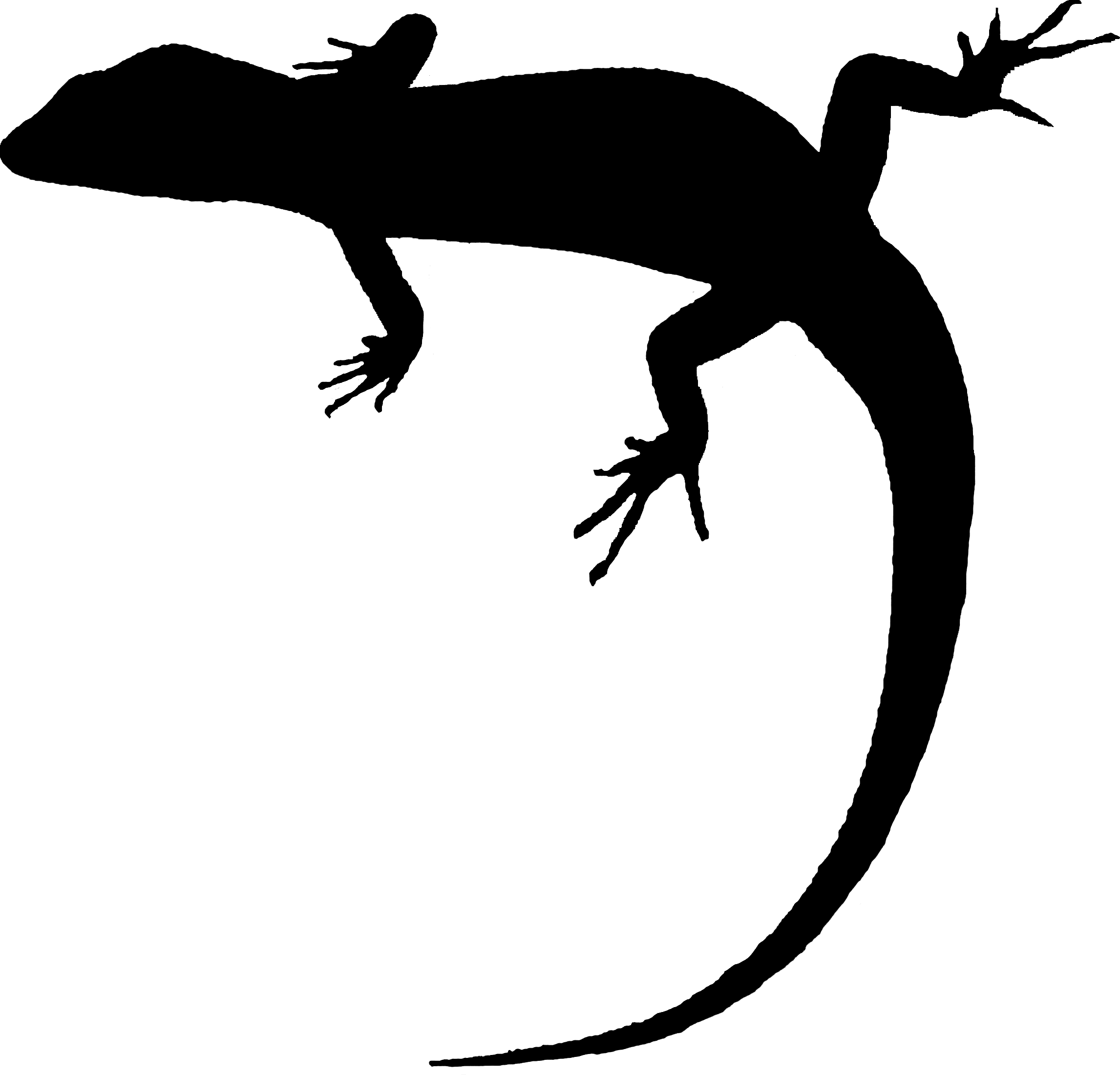 | Threatened | SW  FW | DNA metabar. | Field – U/K | --- | 16S | N/A | | Nine reptile taxa were detected in 10 of 16 sample sites (marine and FW turtles, aquatic and semi-aquatic snakes, and terrestrial skinks) using a metabarcoding assay designed for reptiles. Saltwater crocodile and sea snakes were not detected using eDNA at sites, even though their presence was confirmed visually |
| West et al. 2021b | 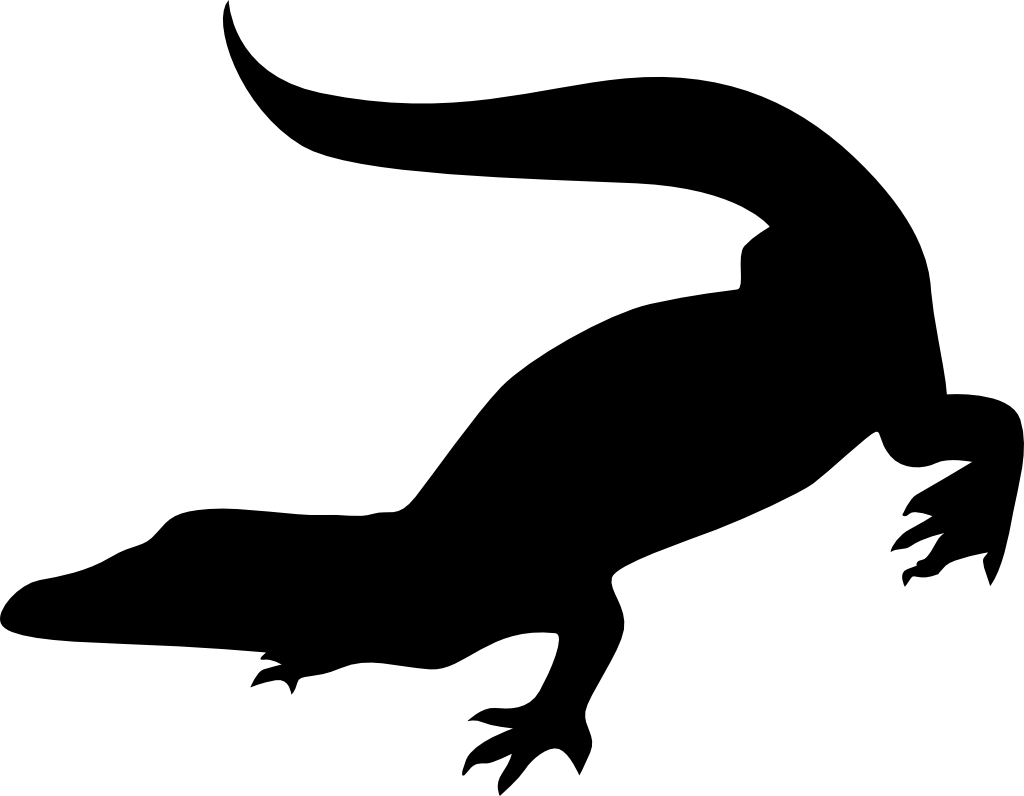  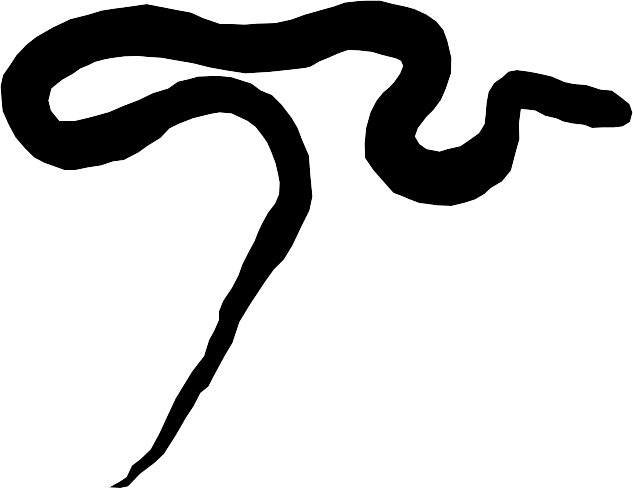  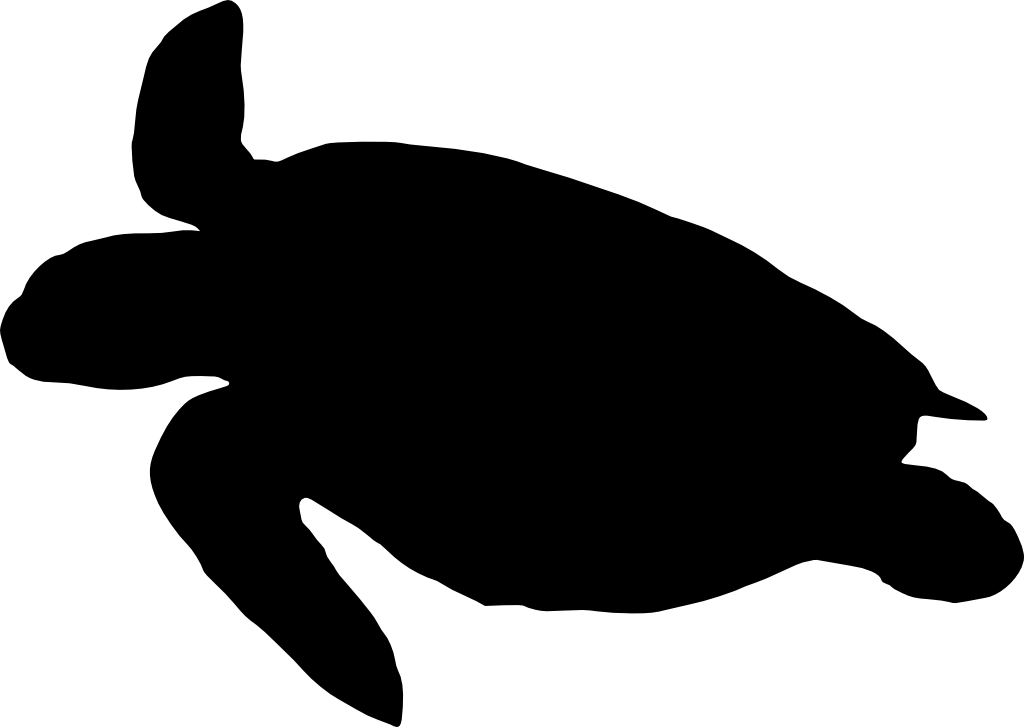 | Threatened  Distribution | SW | DNA metbar. | Field – U | --- | 16S  COI | N/A | | DNA metabarcoding detected five reptile species: saltwater crocodile, black-headed python, Stoke’s sea snake, the white-bellied mangrove snake, and green sea turtle |
| Díaz-Abad et al. 2022 | 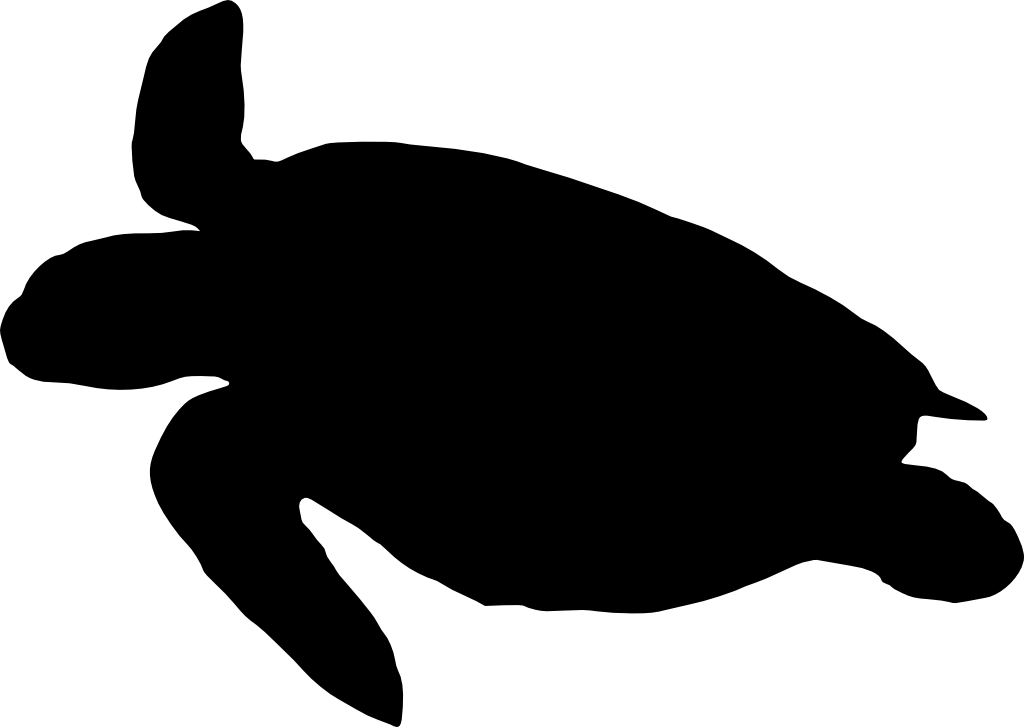 | Threatened  Dietary niche | Swab | DNA metabar. | --- | --- | 18S | N/A | | DNA metabarcoding of esophagus and cloacal swabs of green turtles were validated as an approach for determining turtle diet |
| Lam et al. 2022 | 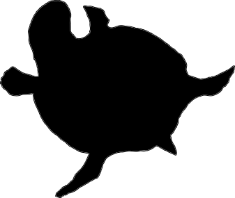 | Threatened | FW  (lotic) | Species-specific | Field – U/K | qPCR | ND4 | TaqMan Exogenous Internal Positive Control | | eDNA sampling was conducted over three years in 34 streams, revealing four new populations of the big-headed turtle (verified by field surveys). |
| Nishizawa et al. 2022 | 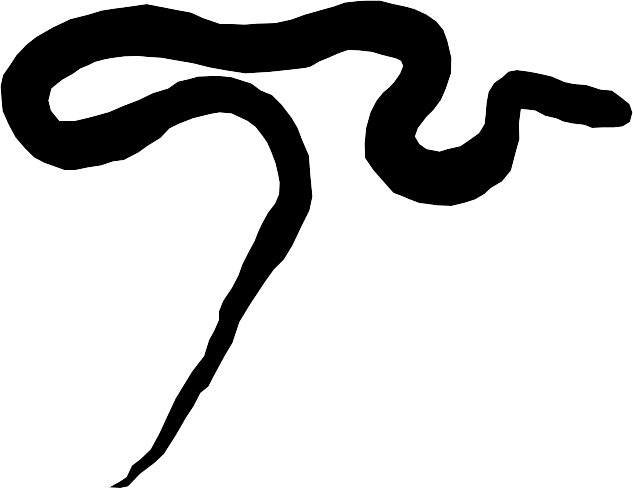 | Ecosystem role | FW  (lentic) | Species-specific | Cont.  Field - U | qPCR | CytB | N/A | | Assays for two of the three snake species (*Rhabdophis tigrinus* and *Gloydium blomhoffii*) were able to detect target DNA in the field |
| Tercel et al. 2022 | 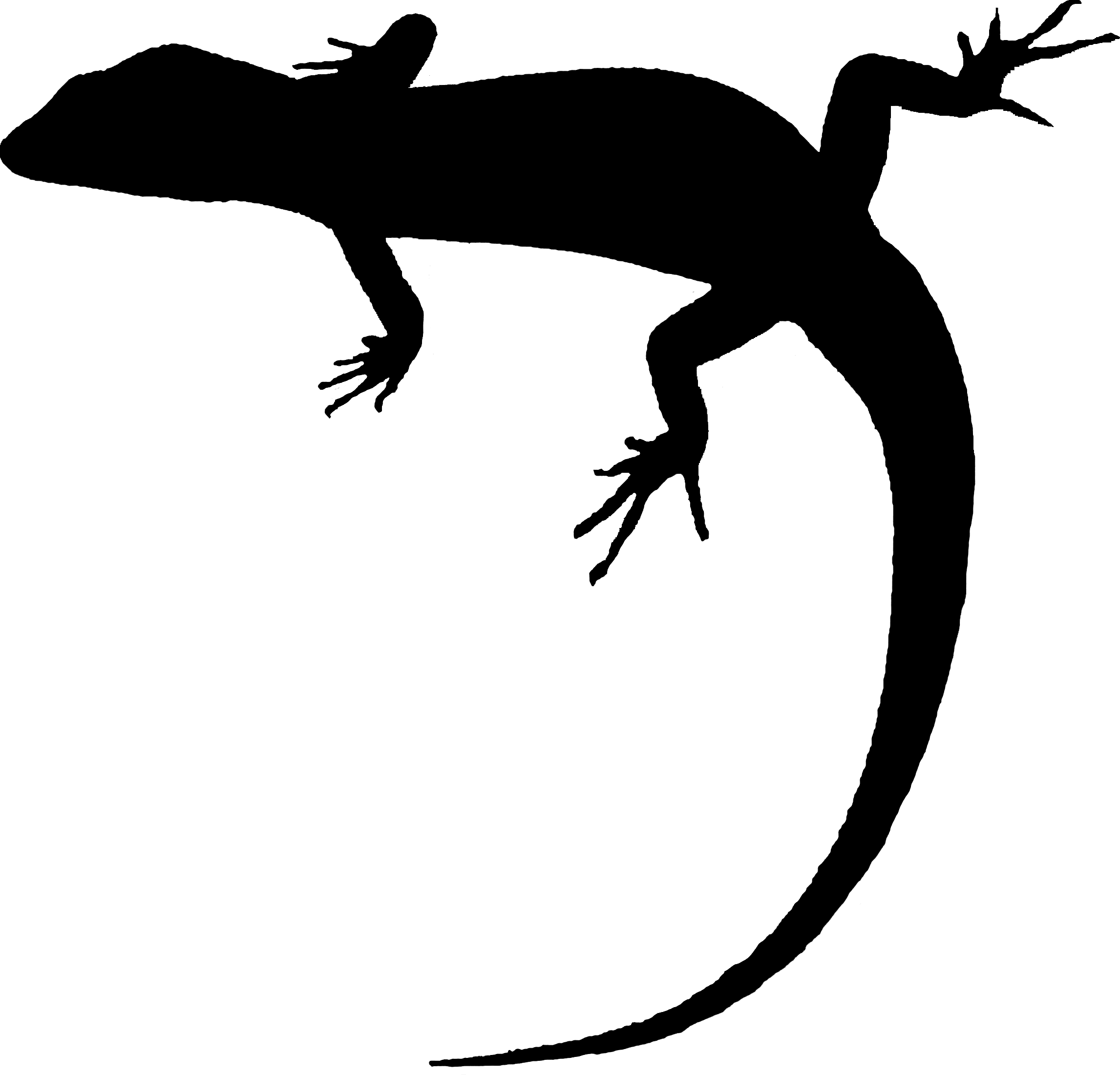 | Threatened  Dietary niche | Faeces | DNA metabar. | --- | --- | COI  ITS2 | N/A | | DNA metabarcoding revealed Telfair’s skink consumes invasive/introduced species frequently. Introduced species made up ~50% of all detection in faecal samples |

(SW = salt water, FW = Freshwater); (lentic = standing water such as lakes and ponds, lotic = running water such as rivers and streams); (Cont. = Controlled setting [ex. Aquarium], Field = sampling only performed in the field, U = presence of target species unknown, K = presence of species confirmed by traditional survey methods); (CR = Control region); (IRK = Inhibition Removal Kit, N/A = information not available).

**REFERENCES (**references for the reptile eDNA studies outlined in Appendix Table 1. If the study was referenced in the main text, the full citation can be found in the reference section at the end of the main text)**:**

Anslan, S., Dalgo, D., Reinhardt, T., Peñafiel, N., Guayasamin, J., Páez-Rosas, D., . . . Steinfartz, S. 2021. DNA metabarcoding reveals fine scale geographical differences of consumed algae in the Galápagos marine iguanas (*Amblyrhynchus cristatus*). *Amphibia-Reptilia*, 42, 1-10. doi:10.1163/15685381-bja10070

Ducotterd, C., Crovadore, J., Lefort, F., Guisan, A., Ursenbacher, S. & Rubin, J.-F. 2020. The feeding behaviour of the European pond turtle (Emys orbicularis, L. 1758) is not a threat for other endangered species. *Global Ecology and Conservation*, 23, e01133. doi:10.1016/j.gecco.2020.e01133

Clare, E. L., Chain, F. J., Littlefair, J. E. & Cristescu, M. E. 2016. The effects of parameter choice on defining molecular operational taxonomic units and resulting ecological analyses of metabarcoding data. *Genome*, 59, 981-990. doi:10.1139/gen-2015-0184

Díaz-Abad, L., Bacco-Mannina, N., Madeira, F. M., Neiva, J., Aires, T., Serrao, E. A., . . . Frade, P. R. 2021. eDNA metabarcoding for diet analyses of green sea turtles (*Chelonia mydas*). *Marine Biology*, 169. doi:10.1007/s00227-021-04002-x

Ducotterd, C., Crovadore, J., Lefort, F., Rubin, J-F., & Ursenbacher, S. 2021. A powerful long metabarcoding method for the determination of complex diets from faecal analysis of the European pond turtle (*Emys orbicularis*, L. 1758). *Molecular Ecology Resources*, 21, 433– 447

Feng, W., Bulté, G. & Lougheed, S. C. 2020. Environmental DNA surveys help to identify winter hibernacula of a temperate freshwater turtle. *Environmental DNA*, 2, 200-209. doi:10.1002/edn3.58

Fyson, V. K. & Blouin-Demers, G. 2021. Effects of landscape composition on wetland occupancy by Blanding’s Turtles (*Emydoidea blandingii*) as determined by environmental DNA and visual surveys. *Canadian Journal of Zoology*, 99, 672-680. doi:10.1139/cjz-2021-0004

Harper, K. J., Goodwin, K. D., Harper, L. R., Lacasella, E. L., Frey, A. & Dutton, P. H. 2020. Finding Crush: Environmental DNA Analysis as a Tool for Tracking the Green Sea Turtle *Chelonia mydas* in a Marine Estuary. *Frontiers in Marine Science*, 6. doi:10.3389/fmars.2019.00810

Kartzinel, T. R. & Pringle, R. M. 2015. Molecular detection of invertebrate prey in vertebrate diets: trophic ecology of Caribbean island lizards. *Molecular Ecology Resources*, 15, 903-914. doi:10.1111/1755-0998.12366

Koizumi, N., Mori, A., Mineta, T., Sawada, E., Watabe, K., & Takemura, T. 2016. Exploratory environmental DNA analysis for investigating plant-feeding habit of the red-eared turtle using their feces samples. *Jurnal Teknologi*, 78, 9-13. doi:10.11113/jt.v78.7253

Kundu, S., Kumar, V., Tyagi, K., & Chandra, K. 2018. Environmental DNA (eDNA) testing for detection of freshwater turtles in a temple pond. *Herpetology Notes*, 11, 369-371.

Lam, I. P. Y., Sung, Y.-H., Lin, L. & Fong, J. J. 2019. Developing quantitative PCR assays to detect threatened and invasive freshwater turtles in Hong Kong using environmental DNA. *Conservation Genetics Resources*, 12, 293-300. doi:10.1007/s12686-019-01103-0

Loeza-Quintana, T., Crookes, S., Li, P. Y., Reid, D. P., Smith, M. & Hanner, R. H. 2021. Environmental dna detection of endangered and invasive species in Kejimkujik National Park and historic site. *Genome*, 64, 172-180. doi:10.1139/gen-2020-0042

Martin, J., Gambaiani, D., Sabatte, M.-A., Pelorce, J., Valentini, A., Dejean, T., . . . Unmack, P. 2021. A comparison of visual observation and DNA metabarcoding to assess the diet of juvenile sea turtle. *Marine and Freshwater Research*, 73, 552-560. doi:10.1071/mf21179

Montoya-Ciriaco, N., Gómez-Acata, S., Muñoz-Arenas, L. C., Dendooven, L., Estrada-Torres, A., Díaz De La Vega-Pérez, A. H. & Navarro-Noya, Y. E. 2020. Dietary effects on gut microbiota of the mesquite lizard *Sceloporus grammicus* (Wiegmann, 1828) across different altitudes. *Microbiome*, 8, 6-6. doi:10.1186/s40168-020-0783-6

Nishizawa, R., Nakao, R., Ushimaru, A. & Minamoto, T. 2022. Development of environmental DNA detection assays for snakes in paddy fields in Japan. *Landscape and Ecological Engineering*. doi:10.1007/s11355-022-00496-9

Orzechowski, S. C. M., Frederick, P. C., Dorazio, R. M. & Hunter, M. E. 2019. Environmental DNA sampling reveals high occupancy rates of invasive Burmese pythons at wading bird breeding aggregations in the central Everglades. *PLoS One*, 14, e0213943. doi:10.1371/journal.pone.0213943

Rajabizadeh, M., Morinière, J. & Rajaei, H. 2021. Adaptation to the hottest spot on earth: Dietary ecology of an enigmatic desert gecko based on DNA metabarcoding. *Journal of Zoological Systematics and Evolutionary Research,* 59, 1470-1480. doi:10.1111/jzs.12558

Rivera, S. F., Vasselon, V., Ballorain, K., Carpentier, A., Wetzel, C. E., Ector, L., Bouchez, A. & Rimet, F. 2018. DNA metabarcoding and microscopic analyses of sea turtles biofilms: Complementary to understand turtle behavior. *PLoS One*, 13, e0195770. doi:10.1371/journal.pone.0195770

Rose, A., Fukuda, Y. & Campbell, H. A. 2020. Using environmental DNA to detect estuarine crocodiles, a cryptic-ambush predator of humans. *Human-Wildlife Interactions*, 14, 64-72.

Schumer, G., Hansen, E. C., Anders, P. J. & Blankenship, S. M. 2019. Development of a quantitative polymerase chain reaction assay and environmental DNA sampling methods for Giant Gartersnake (*Thamnophis gigas*). *PLoS One*, 14, e0222493. doi:10.1371/journal.pone.0222493

Siler, C. D., Freitas, E. S., Yuri, T., Souza, L. & Watters, J. L. 2021. Development and validation of four environmental DNA assays for species of conservation concern in the South-Central United States. *Conservation Genetics Resources*, 13, 35-40. doi:10.1007/s12686-020-01167-3

So, K. Y. K., Fong, J. J., Lam, I. P. Y. & Dudgeon, D. 2020. Pitfalls during in silico prediction of primer specificity for eDNA surveillance. *Ecosphere*, 11. doi:10.1002/ecs2.3193

West, K., Travers, M. J., Stat, M., Harvey, E. S., Richards, Z. T., Dibattista, J. D., . . . Seymour, M. 2021. Large‐scale eDNA metabarcoding survey reveals marine biogeographic break and transitions over tropical north‐western Australia. *Diversity and Distributions*, 27, 1942-1957. doi:10.1111/ddi.13228

Wilson, J. J., Sing, K. W., Chen, P. N. & Zieritz, A. 2018. Tracking the southern river terrapin (*Batagur affinis*) through environmental DNA: prospects and challenges. Mitochondrial DNA Part A: DNA Mapping, Sequencing, and Analysis, 29, 862-866.
